# Holistic shape variation of the rib cage in an adult population

**DOI:** 10.3389/fbioe.2024.1432911

**Published:** 2024-09-18

**Authors:** Andrea Robinson, Bowen Zheng, B. Wade von Kleeck, Josh Tan, F. Scott Gayzik

**Affiliations:** ^1^ Department of Biomedical Engineering, Wake Forest University School of Medicine, Winston-Salem, NC, United States; ^2^ Virginia Tech-Wake Forest Center for Injury Biomechanics, Wake Forest University School of Medicine, Winston-Salem, NC, United States; ^3^ Department of Biomedical Engineering, Columbia University, New York, NY, United States; ^4^ Department of Radiology – Imaging Informatics, Wake Forest University School of Medicine, Winston-Salem, NC, United States

**Keywords:** thorax, anatomical variability, shape variation, medical imaging, automatic segmentation, injury biomechanics

## Abstract

Traumatic injuries to the thorax are a common occurrence, and given the disparity in outcomes, injury risk is non-uniformly distributed within the population. Rib cage geometry, in conjunction with well-established biomechanical characteristics, is thought to influence injury tolerance, but quantifiable descriptions of adult rib cage shape as a whole are lacking. Here, we develop an automated pipeline to extract whole rib cage measurements from a large population and produce distributions of these measurements to assess variability in rib cage shape. Ten measurements of whole rib cage shape were collected from 1,719 individuals aged 25–45 years old including angular, linear, areal, and volumetric measures. The resulting pipeline produced measurements with a mean percent difference to manually collected measurements of 1.7% ± 1.6%, and the whole process takes 30 s per scan. Each measurement followed a normal distribution with a maximum absolute skew value of 0.43 and a maximum absolute excess kurtosis value of 0.6. Significant differences were found between the sexes (*p* < 0.001) in all except angular measures. Multivariate regression revealed that demographic predictors explain 29%–68% of the variance in the data. The angular measurements had the three lowest R^2^ values and were also the only three to have little correlation with subject stature. Unlike other measures, rib cage height had a negative correlation with BMI. Stature was the dominant demographic factor in predicting rib cage height, coronal area, sagittal area, and volume. Subject weight was the dominant demographic factor for rib cage width, depth, axial area, and angular measurements. Age was minimally important in this cohort of adults from a narrow age range. Individuals of similar height and weight had average rib cage measurements near the regression predictions, but the range of values across all subjects encompassed a large portion of their respective distributions. Our findings characterize the variability in adult rib cage geometry, including the variation within narrow demographic criteria. In future work, these can be integrated into computer aided engineering workflows to assess the influence of whole rib cage shape on the biomechanics of the adult human thorax.

## 1 Introduction

Blunt chest trauma, typically caused by motor vehicle crashes (MVCs), falls, contact sports, assault, or occupational incidents, accounts for up to 25% of all trauma related deaths (Edgecombe et al., 2023). Such trauma often results in thoracic injuries, notably rib fractures, which are associated with higher morbidity and mortality compared to cases without thoracic injury (Sirmali et al., 2003; Boele van Hensbroek et al., 2009; Talbot et al., 2017; Benhamed et al., 2022). Due to the significance of blunt chest trauma, much effort has gone into better understanding the mechanisms behind thoracic injuries (Dogrul et al., 2020). Current standards for investigating these injuries include the use of *post mortem* human subjects (PMHSs) (Kroell et al., 1974; Kemper et al., 2011; Lebarbé and Petit, 2012; Kang et al., 2023), anthropomorphic test devices (ATDs) (Xu et al., 2018) and finite element human body models (HBMs) (Gayzik et al., 2011; Vavalle et al., 2014; Kato et al., 2018; John et al., 2022; Robinson et al., 2023) to simulate injurious scenarios. Such experimental or simulation work is intended for regulatory purposes, to elucidate injury mechanisms, or to evaluate potential safety countermeasures to mitigate injury. However, these human surrogates have historically been designed to meet target geometry representing some specific percentile of height and weight (e.g., 50th percentile or “average”), an approach that cannot capture the variation of rib cage shapes present in the adult population. This is an important consideration since research suggests that rib cage shape influences injury risk (Kent et al., 2005; Wang et al., 2016). To accurately assess thoracic injury risk for a more representative share of the population, injury assessment tools like HBMs must move beyond the average geometry and account for the variations within a specific demographic.

Thoracic injury risk is not uniform across populations. In the context of MVCs alone, studies employing post-mortem human subjects, computer models, and field data have indicated an elevated risk of thoracic injury for females, elderly, and obese occupants compared to their male, younger, and non-obese counterparts (Bose et al., 2011). In comparable crash scenarios, belted female drivers were 38% more likely to sustain a chest Abbreviated Injury Scale (AIS) 2+ injury than belted male drivers (Bose et al., 2011). Despite the safety advances of modern vehicles, females still have significantly higher risk of injury, with odds ratios of 1.56 and 2.14 higher odds of AIS 2+ and AIS 3+ rib fracture, respectively (Forman et al., 2019). Field data analyses further indicate that obese occupants had a 33% higher risk of AIS 3+ thoracic injury compared to non-obese occupants (Cormier, 2008), a result later confirmed with computational tests where human finite element models representing different obesity levels predicted higher risks of thoracic injury (Shi et al., 2015; Gepner et al., 2018). When considering staircase falls, older individuals were more susceptible to rib fracture than children, but males experienced more thoracic injuries than females (Boele van Hensbroek et al., 2009). In these common trauma scenarios, there is a need to elucidate the mechanisms that make some more susceptible to injury over others.

The variation seen in thoracic response has partly been attributed to differences in geometric properties of the rib cage which affect the rib cage’s ability to absorb and respond to external forces. Thus, prior research has set out to characterize the structural response and shape of individual ribs. Numerous studies have shown that the structural properties of individual ribs are affected by rib level, cross sectional geometry, and overall size of the rib (Agnew et al., 2018; Fleischmann et al., 2020). Rib geometry has been characterized with arcs (Schultz et al., 1974), a combination of a circle and a semi-ellipse (Kindig and Kent, 2013), and more recently logarithmic spirals (Holcombe et al., 2016).

An alternative approach has been to study the rib cage on a more holistic scale and use the whole rib cage. In real-world blunt chest trauma, ribs are not impacted in isolation, and analysis of whole rib cage geometry may better represent characteristics that influence injury risk. These studies often utilize landmarks collected through manual placement (Gayzik et al., 2008; Weaver et al., 2014; Wang et al., 2016) or landmarks derived from individual rib shape models (Larsson et al., 2022) to represent the rib cage. Morphological techniques such as Principal Component Analysis (PCA) or Generalized Procrustes Analysis (GPA) are then used to determine predicted landmark locations. However, the resulting models are useful for predicting a single average anatomy for a given set of demographic parameters and do not consider unique phenotypes differing from this average. Given scatter in the reported data, relying solely on the average anatomy may yield inaccurate predictions for individual outcomes. A recent study sought to address these limitations by quantifying the variability in rib cages among 89 adult males aged 18 years and older using the standard deviations of individual principal component (PC) scores (Larsson et al., 2022). However, this dataset includes no females, is limited to a narrow range of statures and weights, and bases rib cage shape on PCs, which inherently limits the interpretability of the results in terms of the original data. Further, prior investigations have been limited in sample size due to the time constraints of manual segmentation and analysis. While PCA helps reveal the underlying patterns that contribute to morphological diversity, we aim to provide a more readily interpretable characterization of rib cage geometries in an adult population.

Therefore, this study aims to achieve a holistic characterization of rib cage variation within an adult population. A fully automated pipeline is presented to analyze the variability in rib cage shape within adults aged 25–45 ears old. Utilizing a machine learning (ML) based segmentation tool (Wasserthal et al., 2023), 3D rib cage reconstructions are extracted, and rib cage measurements are collected from the reconstructions. Distributions of these measurements, based on a sample of 2,250 scans, are provided. The result of this study is a dataset of real-world rib cage data intended for use in CAE pipelines for the study of thoracic biomechanics.

## 2 Materials and methods

### 2.1 Data source

**FIGURE 1:**
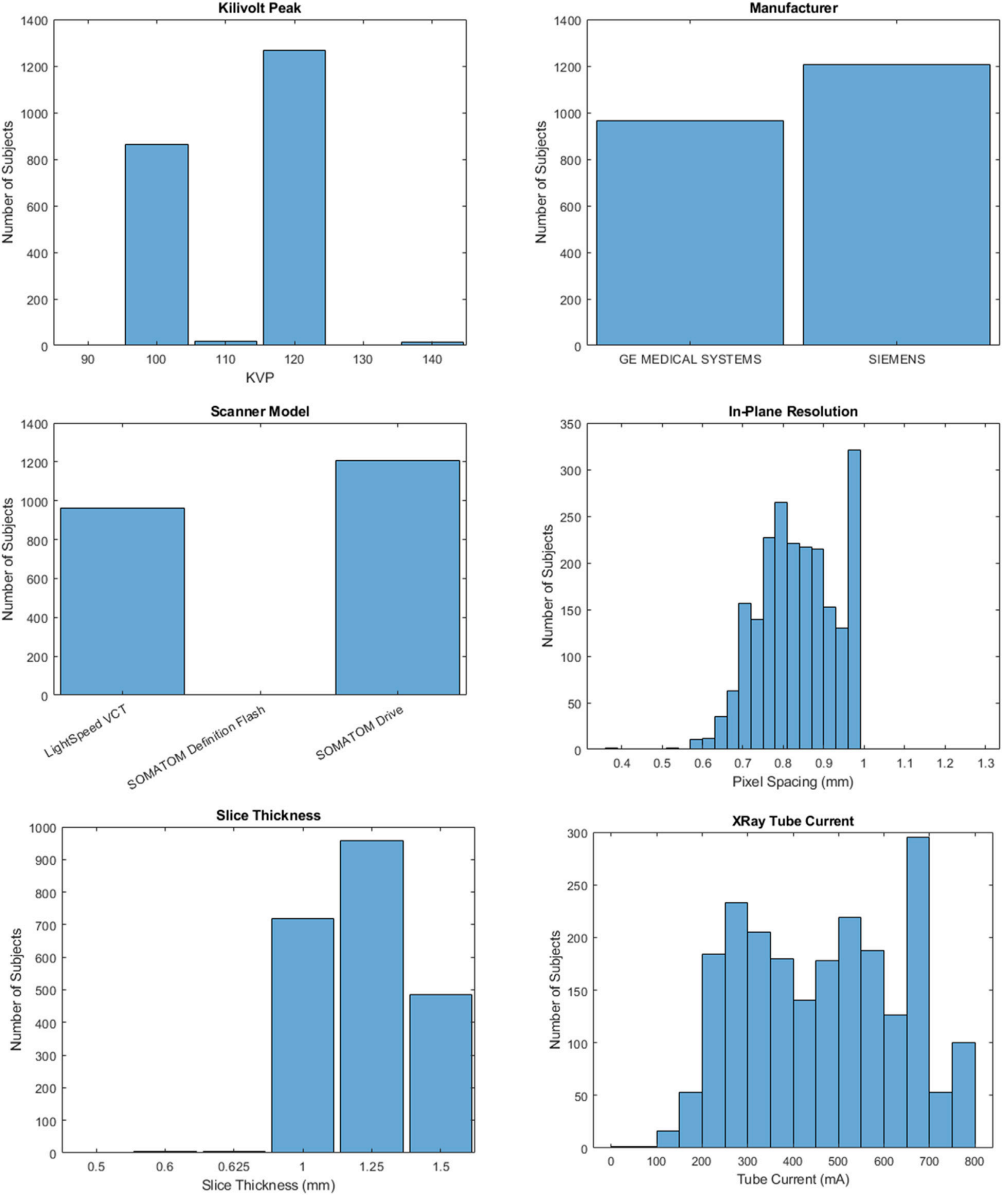
Distribution of study cohort scan characteristics.

**FIGURE 2:**
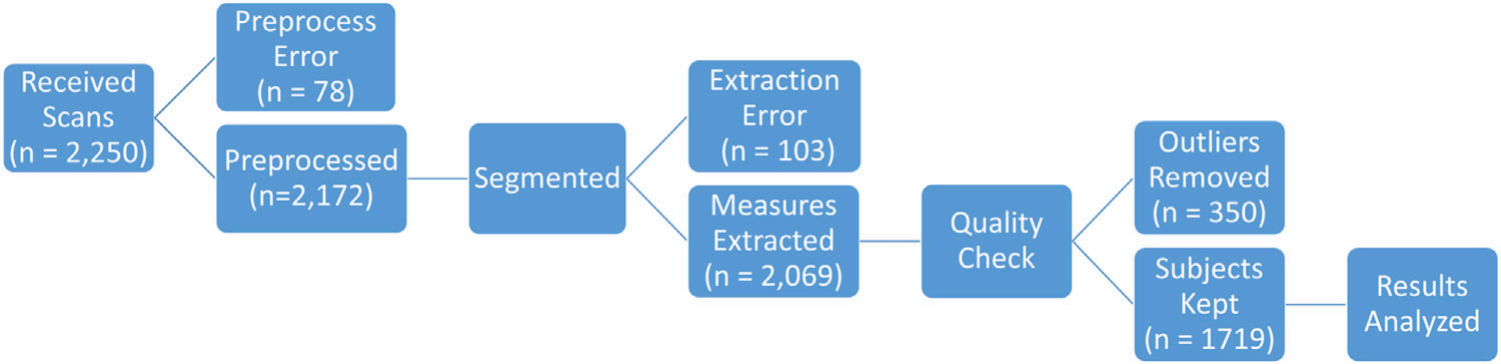
Overview of analysis pipeline.

After obtaining IRB approval through Wake Forest’s Institutional Review Board (#IRB00006511), 2,250 Chest-Abdomen-Pelvis computer-aided tomography (CT) series with contrast were obtained from patients of Atrium Health Wake Forest Baptist Hospital between 2016 and 2023. CTs were performed on either GE or Siemens manufactured scanners. Slice thickness varied between 0.5 and 1.5 mm while in-plane resolution varied between 0.365 and 1.270 mm. Further details about the CT scans are included in Figure 1. All patients were aged 25–45 at the time of the scan. CT series were converted from DICOM to NIFTI using the dicom2nifti python library (Brys). Series where conversion failed (n = 78) or without all 12 rib pairs in the scan field of view (n = 103) were excluded (Figure 2).

### 2.2 Auto-segmentation method

We utilized the open-source deep learning segmentation model TotalSegmentator (Wasserthal et al., 2023) to generate segmentations of the rib cage and sternum for shape analysis. A single NIFTI file was saved containing individual labels for each rib. A separate NIFTI file was saved containing the sternum segmentation.

### 2.3 Comparison to manual segmentations

The developers of TotalSegmentator previously evaluated segmentation quality using the Dice similarity coefficient, which quantifies how similar an image segmentation output is to its ground truth on a scale of 0–1. They achieved a score of 0.96 out of 1 for the rib class. However, to evaluate whether the TotalSegmentator results were adequate for the rib cage shape analysis in this study, we performed an independent evaluation of segmentation quality.

Manual segmentations from a prior study on rib cage shape changes with age (Weaver et al., 2014) were compared to their corresponding automated segmentations produced by TotalSegmentator. A total of 26 subjects aged 20–50 were compared. First, a best fit alignment in Geomagic Wrap 2021 (v2021.2.13; 3D Systems, Morrisville, NC, United States) aligned the corresponding segmentations to ensure both segmentations were in the same orientation. The 3D compare tool was then used to calculate the surface deviation between the segmentations. Maximum/minimum deviation was set to ±10 mm, and the tolerance was set to ±1.25 mm. Outcome measures included average positive deviation, average negative deviation, standard deviation, and root mean square (RMS) estimation. An exemplar deviation spectrum is provided in Supplementary Figure S1.

### 2.4 Measure extraction

Ten measurements of rib cage shape were collected from each scan in a fully automated process using custom MATLAB code. The output of TotalSegmentator, a binary mask, was first converted to a point cloud. The rib cage positioning was then normalized across all scans, ensuring that the most posterior points, commonly referred to as the angles of the ribs, on both the left and right sides were located in the same plane. While the scanner bed largely normalized positioning, this initial step accounts for differences in patient positioning that could lead to overestimation of rib cage measurements and is depicted in Supplementary Figure S2.

#### 2.4.1 Measure definitions

**FIGURE 3:**
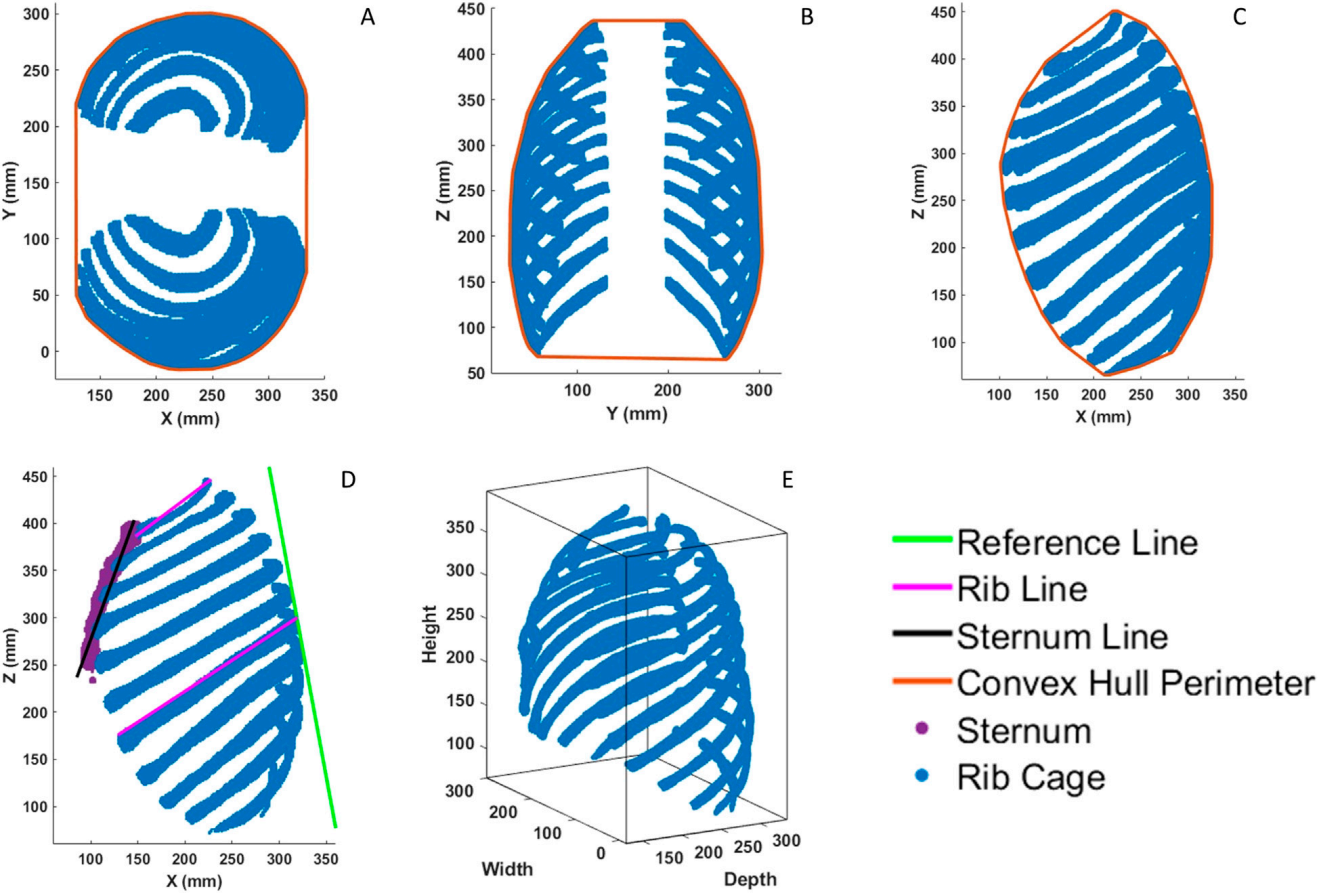
**(A)**, Axial convex hull area measurement. **(B)**, Coronal convex hull area measurement. **(C)**, Sagittal convex hull area measurement. **(D)**, Lines used in angular measurements. Angles are measured with respect to the green reference line. **(E)**, Bounding box used for height, width, and depth measurements.

The ten measures selected to characterize rib cage shape were height, width, depth, convex hull areas in the axial, sagittal, and coronal planes, convex hull volume, rib 1 and rib 7 angles, and sternum angle (Figure 3). These measures were chosen to encompass biomechanically relevant features of the whole rib cage. Chest deflection is the current standard for developing thoracic injury risk curves in both frontal (Mendoza-Vazquez et al., 2015) and lateral (Humm et al., 2021) impacts and are normalized by overall width and depth. The convex hull areas can be related to common planes of impact in blunt trauma and provide a fuller understanding of how forces are distributed across the rib cage, particularly in oblique impacts (Humm and Yoganandan, 2020). Lastly, rib angle is known to affect the stiffness of the rib cage and influence injury risk (Kent et al., 2005).

Rib cage dimensions were calculated on the point cloud using a minimally fitting bounding box (i.e., the difference between the minimum and maximum value in each coordinate) as shown in Figure 3E. Convex hull areas were calculated on a 2D projection of the 3D point cloud in the axial, coronal, and sagittal planes (Figures 3A–C).

Angular measurements were found using the standard trigonometric equation for computing angle between two lines given their slopes. The lines used were a common reference line defined for each rib cage and a single line representing either rib 1, rib 7, or the sternum. Exemplar lines are provided in Figure 3D for reference. It is important to note that “rib angle” in this study refers to the angle as measured in the sagittal plane and defines the “pump-handle” motion of the rib cage. This is not to be confused with the anatomical landmark “angle of the rib”. Similarly, “sternum angle” in this study refers to the angle of the whole sternum as measured in the sagittal plane, and not the anatomical landmark “sternal angle” which represents the angle difference between the manubrium and sternal body.

This reference line was defined as a line fit to the most posterior point on ribs 6–8. This was opposed to using the global *Z*-axis which does not account for differences in spinal curvature. For rib angles, the rib line was defined to best match the methodology presented by Kent et al. (2005) in which a line connecting the superior-most posterior point of the rib to the superior-most anterior point of the rib was used. For sternum angles, the sternum line was found by using PCA on the sternum point cloud. The first two principal components (PCs) were used to fit a plane to the sternum point cloud. The slope of that plane in the XZ plane, or the depicted sternum line of Figure 3D, is derived from the first PC and represents the direction of greatest variance of the sternum.

#### 2.4.2 Validation of measurement techniques

Manual measurements were collected on the 3D segmentations of five randomly selected subjects using the open-source software 3D Slicer (Fedorov et al., 2012) to confirm the accuracy of the automated measure extraction process. These hand measures were taken to mimic the automated pipeline, and any measures that differed by more than 5% were flagged for refinement of the measurement methodology.

#### 2.4.3 Outcome measures

The outcome measures consist of distributions of each rib cage measurement provided in histograms and scatterplots vs. body mass index (BMI) by sex. The scatterplots also contain 95% confidence interval ellipses to give a visual representation of the variability associated with estimating the mean of the data. The ellipses, centered on the means of the data, reflect the covariance between the rib cage measure and BMI, with elongated shapes indicating higher correlation and larger sizes indicating higher variability. Students t-tests were performed to compare female and male measures at a significance level of α = 0.01.

A multivariate multiple regression was performed to examine how multiple demographic predictor variables simultaneously related to multiple response variables. The demographic predictors included age, stature, weight, BMI, and sex. Correlations between these five variables were calculated and can be found in Supplementary Figure S3. Given the high correlation between weight and BMI, Akaike Information Criterion (AIC) and Bayesian Information Criterion (BIC) were used to determine which of those two predictors to keep in the final model. AIC and BIC were computed for models including either weight or BMI, and the predictor that resulted in lower AIC and BIC values, indicating a better model fit, was selected. The significance of each predictor variable was evaluated using t-tests, with a significance level set at α = 0.01, and adjusted R-squared values were reported to assess the explanatory power of the model. Relative importance of each demographic predictor was calculated using the relaimpo package in R-studio (Groemping, 2006). The resulting importances for each rib cage measurement were normalized to sum to 100%.

## 3 Results

### 3.1 Segmentation quality

All scans that were run through TotalSegmentator successfully produced an output NIFTI file containing its segmentation. Qualitative assessment revealed that the posterior portion of the ribs from the head to the costotransverse joint were excluded from the segmentations. This result was expected, however, because TotalSegmentator was not designed to capture this portion of the rib. The most posterior aspect of the ribs were needed for the measurements collected in this study, and were present in the segmentations. Comparing manual and automated segmentations from the separate subset of 26 subjects showed that the average positive and negative deviation was within ±0.5 mm, with an average standard deviation of 0.64 mm and RMS of 0.63 mm. The full set of data from this analysis is provided in Supplementary Table S1. Overall, the difference between manual and automated segmentation methods was within the scan resolution, and the main portion of the rib required to obtain the rib cage measures was present in the segmentations.

Within the main set of 2,069 rib cage segmentations, some were removed after a manual quality check revealed outliers in the dataset (Figure 2). Outliers included scans in which non-rib artifacts remained in the segmentation (3% of scans) and scans where ribs were mislabeled (17% of scans). A total of n = 350 scans were excluded. The same author completed this step in a time frame of 3.5 h, or 6 s per scan.

### 3.2 Final study population

**FIGURE 4:**
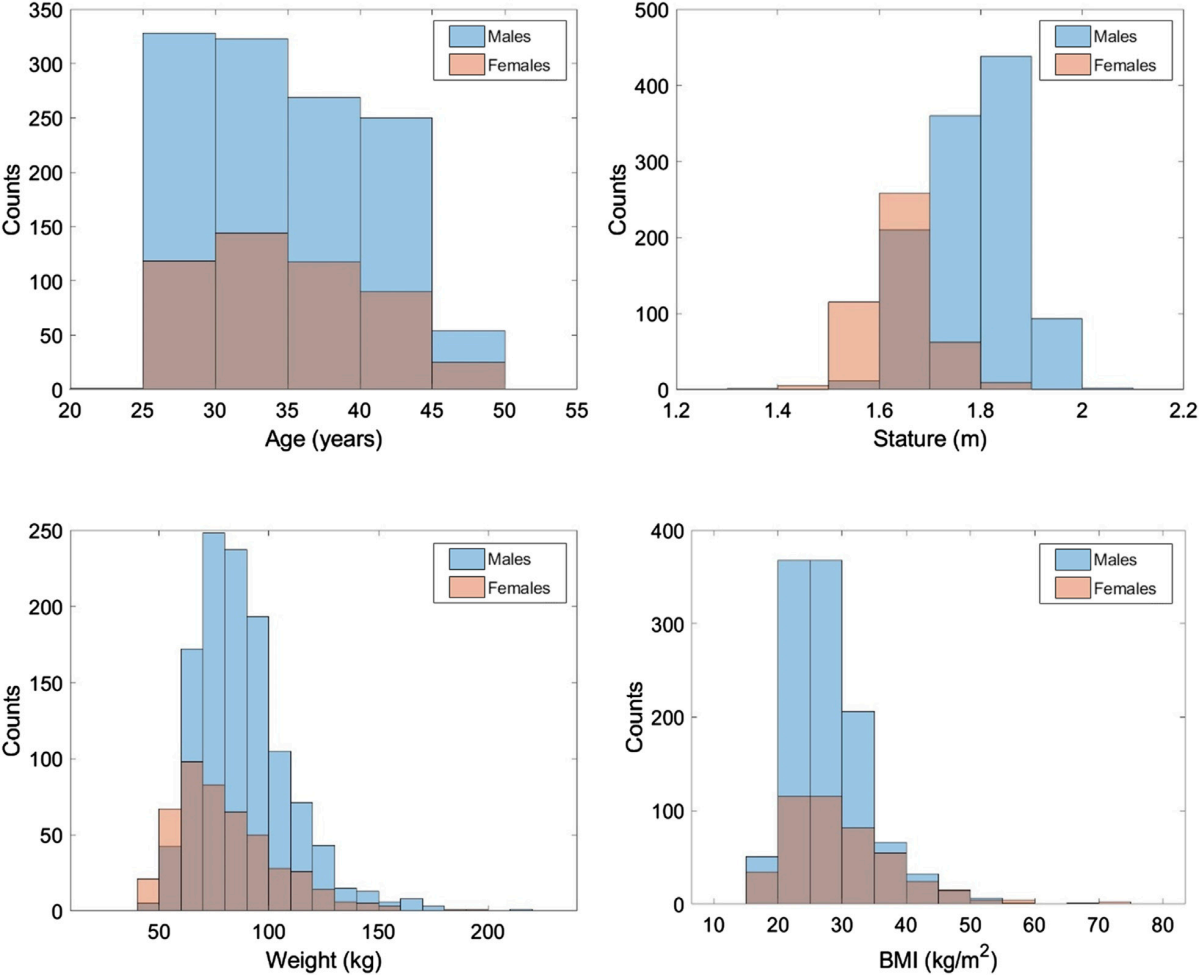
Study population histograms. Male and female distributions are overlayed.

After all exclusions, the final sample size was 1719 subjects. Demographics of the study population are provided in Figure 4 with male and female distributions overlayed. The associated means and standard deviations are provided in Table 1. BMI was not available in the medical record for 9% of subjects (44 females and 112 males). For results involving BMI, those subjects were excluded.

**TABLE 1:** Means and standard deviations for study population demographics. Column of BMI had a smaller sample size due to lack of data for all subjects.

	Age (years)	Stature (m)	Weight (kg)	BMI* (kg/m^2^)
**Females (n = 494)** (*n = 450)	34.4 ± 6.0	1.64 ± 0.08	80.35 ± 23.70	29.89 ± 8.62
**Males (n = 1,225)** (*n = 1,113)	34.4 ± 6.1	1.78 ± 0.08	88.33 ± 21.58	27.72 ± 6.24

### 3.3 Distributions of rib cage measurements

#### 3.3.1 Measurement validation

The results of the measurement technique validation are shown in Table 2. For the 5 subjects evaluated, the automated output deviates from manual measurements by less than 3% on average, indicating the pipeline accurately calculates measures of interest.

**TABLE 2:** Average percent difference between automated and manual measurements of rib cage shape.

	Height	Width	Depth	Coronal area	Sagittal area	Axial area
Average % Difference	1.1 ± 1.0	1.0 ± 1.2	2.1 ± 1.7	1.4 ± 1.3	1.4 ± 2.1	2.9 ± 2.0

#### 3.3.2 Distributions

**FIGURE 5:**
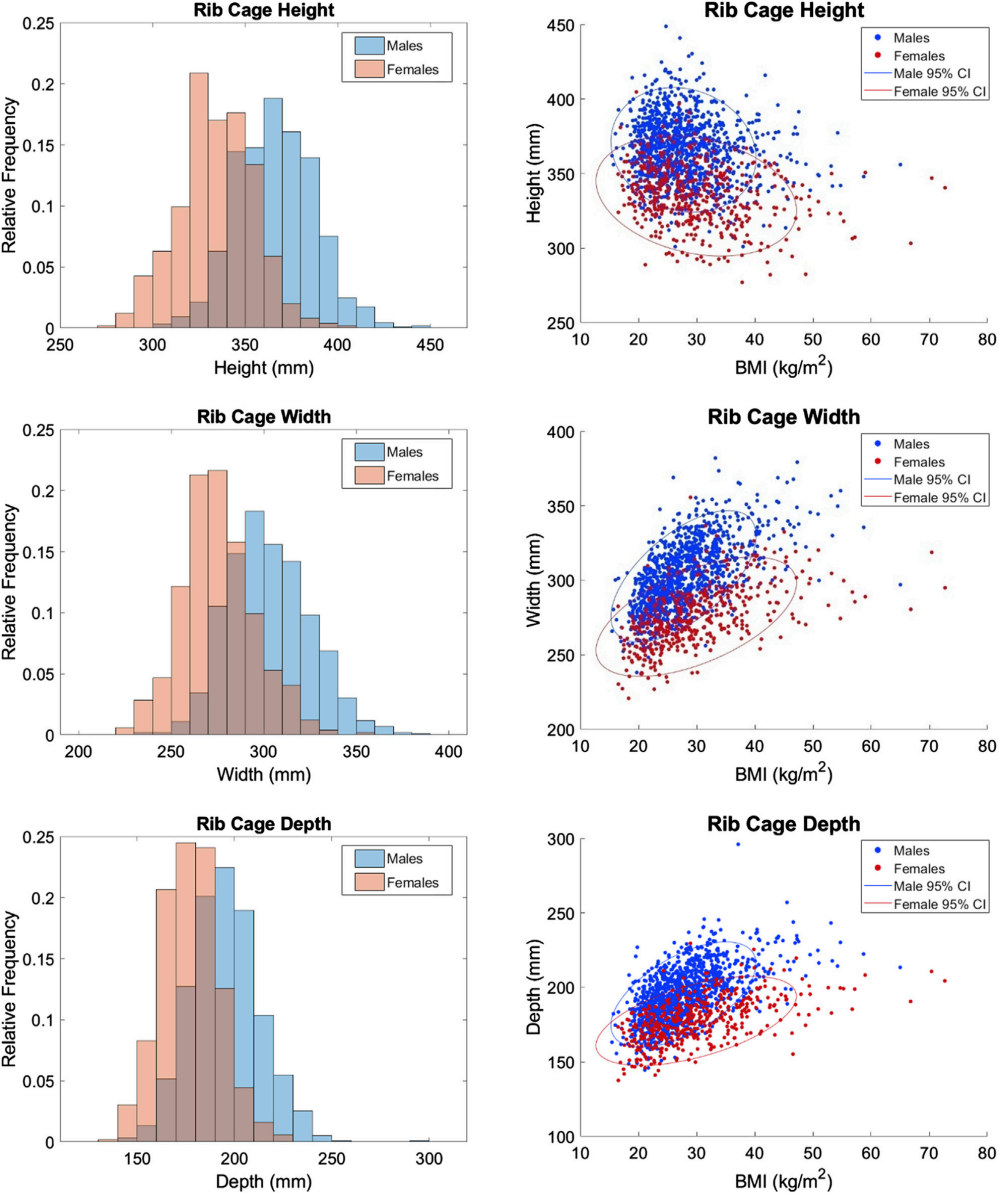
Distributions of dimensional measurements vs. BMI.

**FIGURE 6:**
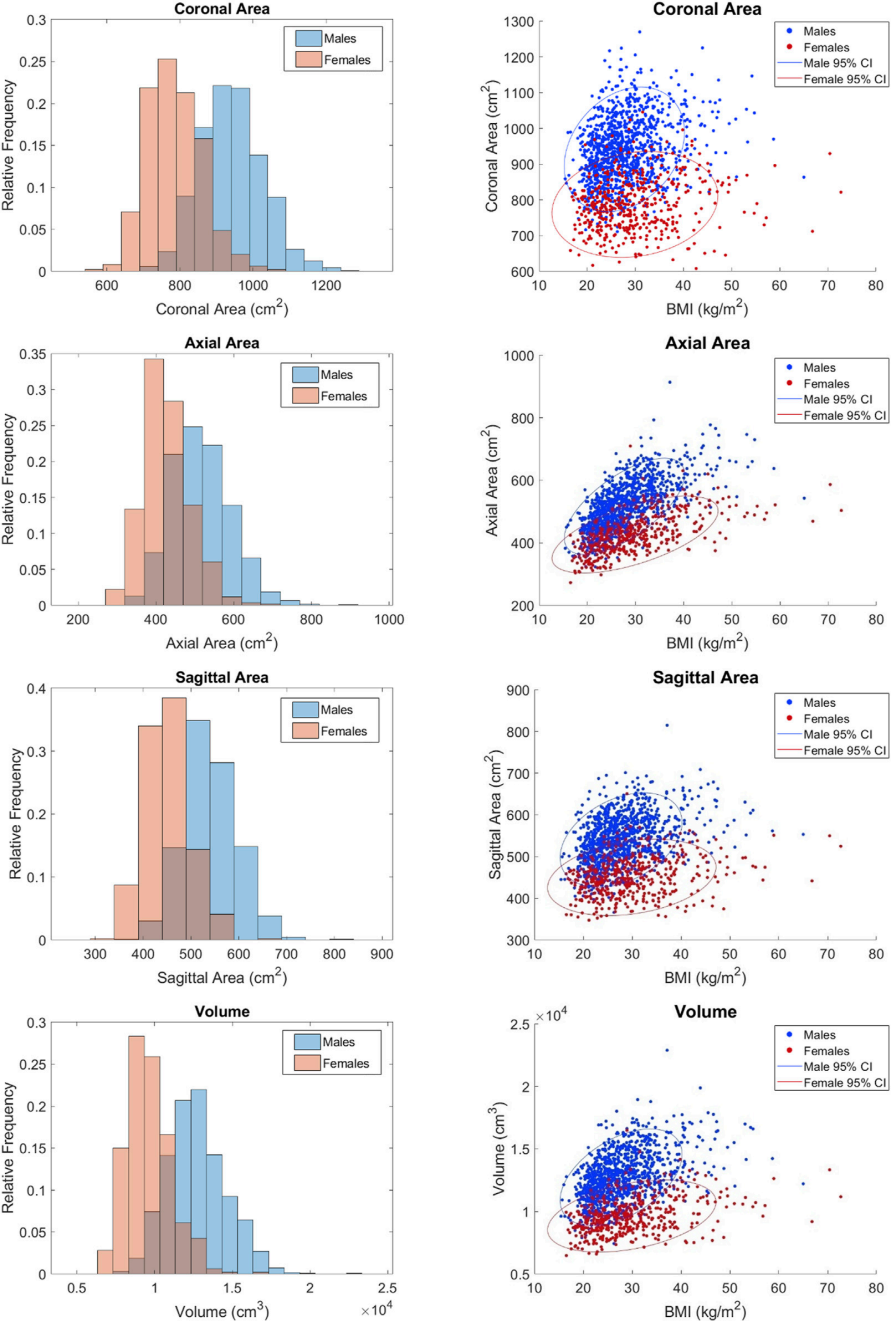
Distributions of area and volumetric measurements vs. BMI.

**FIGURE 7:**
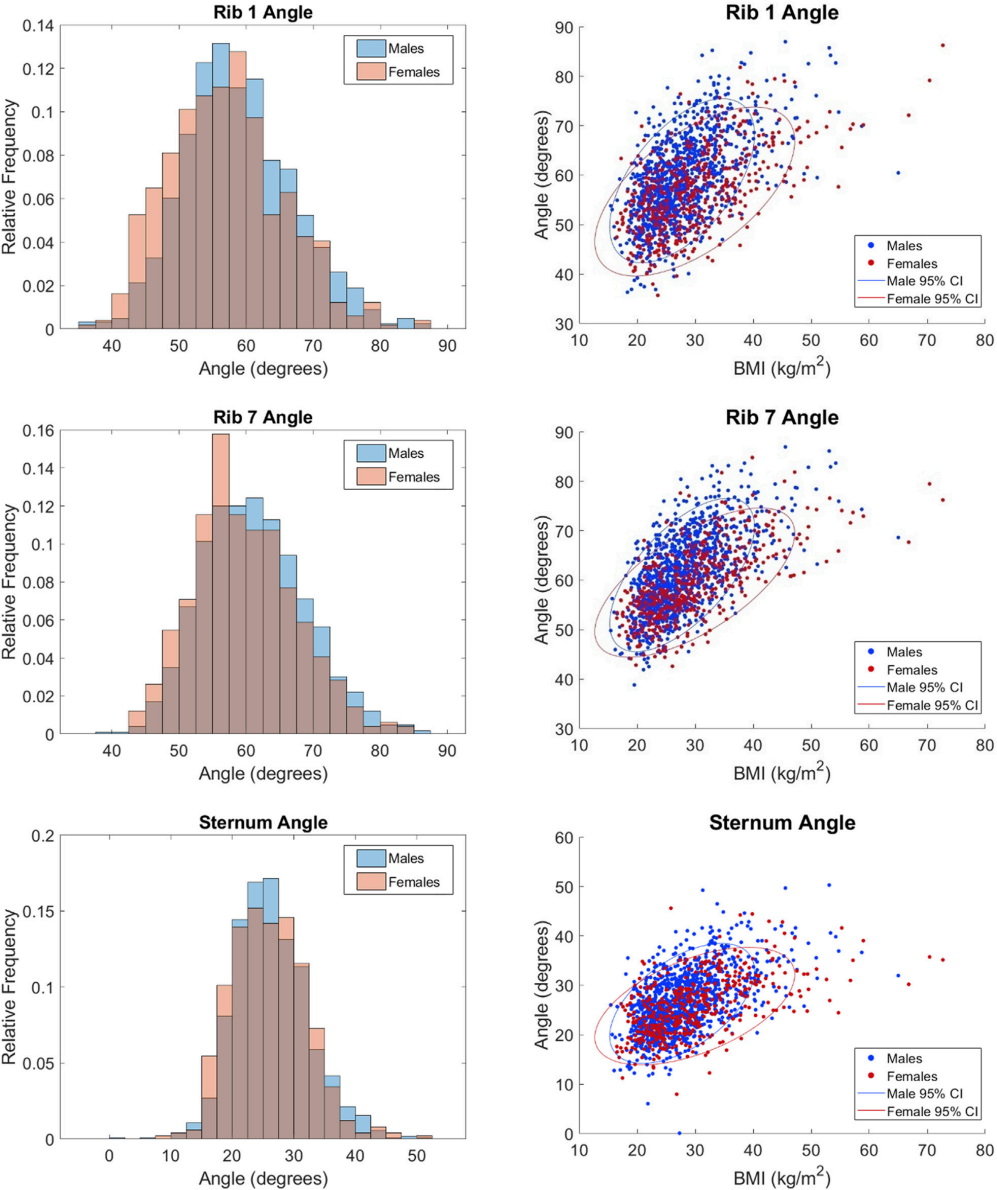
Distributions of angular measurements vs. BMI.

The distributions of each measurement in histograms by sex and scatterplots vs. BMI by sex are shown in Figures 5–7. All measures were found to follow a normal distribution based on the requirements established for clinical data (Kim, 2013) and had skewness values ranging from −0.09 – 0.43 and kurtosis (excess) values ranging from −0.29 – 0.6.

Statistically significant differences were found between the sexes for all measures except sternum angle. For example, male ribs cages are 27.35 mm wider on average than female rib cages. Descriptive statistics for the full dataset are provided in Table 3. The difference between the sexes is less obvious in angular measurements where there is no clear distinction in their respective distributions. This is consistent with the nondimensionality of angle measures. Upon closer examination, the difference between means was 2.0°, 1.5°, and 0.4° for rib 1 angle, rib 7 angle, and sternum angle, respectively. Despite the statistical tests showing significance, the observed differences are not likely to be clinically significant.

**TABLE 3:** Descriptive Statistics of Sample by sex.

	Mean		STD Dev		Min		Max	
Variable	Female	Male	Female	Male	Female	Male	Female	Male
Height (mm)	334.30	365.88	20.23	20.92	277.00	301.00	404.80	448.80
Width (mm)	275.43	302.78	19.82	21.91	220.78	237.97	355.69	382.07
Depth (mm)	177.66	195.32	15.17	17.52	137.57	144.58	229.60	296.08
Coronal (cm^2^)	785.33	940.79	73.69	86.79	585.27	711.05	1,045.59	1,269.64
Sagittal (cm^2^)	449.63	539.94	46.28	56.19	337.90	366.17	650.67	814.92
Axial (cm^2^)	426.69	516.96	61.88	76.37	272.81	322.85	709.49	913.64
Volume (cm^3^)	9631.29	12,733.18	1,453.35	1919.73	6340.39	7428.89	16,485.45	22,885.75
Rib 1 Angle (degrees)	56.97	58.93	8.53	8.23	35.71	36.34	86.25	86.99
Rib 7 Angle (degrees)	59.67	61.13	7.55	7.67	42.59	38.82	84.79	86.95
Sternum Angle (degrees)	26.01	26.41	6.11	6.06	7.97	0.09	50.46	50.29

For completeness, scatterplots vs. age, stature, and weight are provided in Supplementary Figures S4-S6. BMI alone tends to have a larger effect on males than females as indicated by the steeper orientation of the principal axis in the confidence ellipses for males. This may be attributed to the higher BMIs seen in the female subjects. Rib cage height was the only measurement that had a negative correlation with BMI per Figure 5.

### 3.4 Predictive models of rib cage geometry

Although the focus of this study was on providing a robust dataset of rib cage geometries, multivariate multiple regression was performed to highlight the limitations of using demographic information to predict rib cage geometry in the face of such large phenotypic variation. Weight was used as a predictor in the final model over BMI because it produced a model with lower AIC and BIC scores, so the final predictors were age, stature, weight, and sex. The proportion of variance explained by each predictor is provided in Table 4, and the full table of model regression coefficients is supplied in Supplementary Table S2. As shown, demographic predictors only account for 29%–68% of the variance seen in the rib cage measurements. The smallest R^2^ values were seen for angular measurements indicating that demographic factors may not be most influential in determining rib and sternum angles. Higher dimensional measures account for a greater share of the variance, with volume having the highest. Age was insignificant for rib cage height. All other predictors were significant for all measurements.

**TABLE 4:** Estimates of model fit.

	Residual standard Error	Adjusted R-squared
Height (mm)	17.49	0.51
Width (mm)	15.83	0.59
Depth (mm)	12.67	0.54
Coronal (cm^2^)	65.52	0.64
Sagittal (cm^2^)	40.69	0.63
Axial (cm^2^)	49.56	0.65
Volume (cm^3^)	1,291.94	0.68
Rib 1 Angle (degrees)	6.82	0.34
Rib 7 Angle (degrees)	5.86	0.42
Sternum Angle (degrees)	5.07	0.29

**FIGURE 8:**
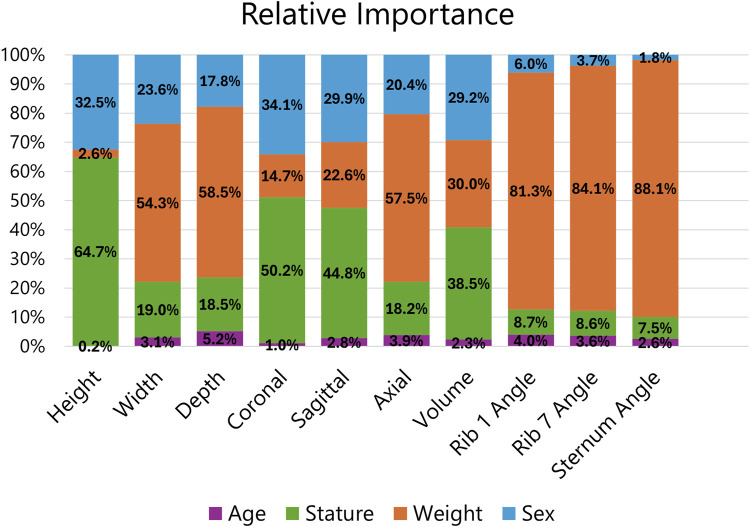
Relative importance of demographic predictors on rib cage measurements.

The relative importance of the demographic predictors on the rib cage measurements is provided in Figure 8. The regressions found that most predictors were significant for all measurements, but their contribution to each measure varies. Age consistently had the smallest relative importance with an average of 2.9%, but the importance of stature, weight, and sex depended on the measurement. Subject weight was the dominant demographic factor for angular measures, surpassing 80%, but had little influence on rib cage height.

## 4 Discussion

In this study, we aimed to characterize the variability in rib cage shape seen within an adult population sample. To address the limitations of manual segmentation on sample size, we employed a ML-based automatic segmentation tool, establishing an automated pipeline for the consistent and accurate extraction of rib cage measurements. Our findings revealed that each measured parameter exhibited a normal distribution, supporting the robustness of the ML-based automated segmentation method in producing high-quality rib cage segmentations for variability studies. The resulting methodology curated a dataset representing real-world rib cages in an adult population and categorized them according to their percentiles within the dataset. This dataset facilitates the generation of multiple rib cage geometry targets that align closely with a narrow range of subject demographics.

To our knowledge, this is the first study to characterize whole rib cages using automatically generated segmentations at a scale of over 1700 samples. The study cohort contained CT scans with a diverse set of scan characteristics. The automated segmentations were suitable for geometric analysis as evidenced by their similarity to manually segmented ground truths. Due to the large sample size, we developed a fully automated methodology to extract rib cage measurements. This has benefit over methods involving manual landmarking which are time consuming to obtain and susceptible to user variability. The methods here required on the order of seconds to produce a segmentation and to extract all ten measurements.

All measurements were initially plotted against BMI for multiple reasons. First, it is often used as an indicator of health (Khanna et al., 2022) and is associated with pooerer outcomes in trauma cases. Second, it encompasses the stature and weight predictors. Lastly, the measurements were not likely to change with age due to the narrow age range of this study and past research indicating low explanatory power of age on variance in rib cage shape (Kent et al., 2005; Gayzik et al., 2008; Holcombe et al., 2017). Although Figure 5 indicates that angular measures have a positive correlation with BMI, Supplementary Figures S5-S6 reveal that this is largely driven by weight and not stature, a result consistent with Kent et al. (2005). The remaining measures had similar correlations between BMI, stature, and weight.

The methodology for collecting rib cage height, width, and depth is an extension of the methods proposed by Lynch et al. (2023) which utilized a bounding box to obtain rib cage dimensions from 141 subjects aged 10–80 years old. To compare data from the current study to Lynch et al. we looked at subset of that data from within 30–40 years (n = 20). Our results are greater than these values, with averages for height, width, and depth of 356.8 ± 25.2 mm, 294.9 ± 24.7 mm and 190.2 ± 18.7 mm compared to 341.6 ± 26.0 mm, 283.4 ± 27.0 mm, and 184.2 ± 15.0 mm. However, the sample size in the current study is 86 times larger than this subset of adults and likely encompasses a broader range of individuals.

Rib angles are a common measurement of interest given their impact on how force is transmitted through the chest. This angle dictates the component of a force vector acting normal to the plane of a rib which influences how much force is experienced. Sternum angle is important for similar reasons. In this study, we found a high degree of variability in all angular measurements as noted by the lower adjusted R^2^ values. From Supplementary Figure S8, we saw that rib 7 angle can vary as much as 13° between individuals of similar height and weight. Sex differences were not apparent as the distributions of angles largely overlapped between the sexes unlike the non-angular measurements. The lack of sex differences is further demonstrated by its low relative importance on angular measurements from Figure 8, which did not surpass 6%. The regression model predicts a 4° difference in rib 7 angle between males and females of the same demographic. This is compared to literature values of 1.03° (Kent et al., 2005), 3.0° (Holcombe et al., 2017), and 3.77° (Holcombe et al., 2020).

While versions of the extracted measurements can be found in literature, convex hull areas have not been employed in previous discussions regarding whole rib cage shape. These measurements serve as an analog to the plane of impact experienced in blunt trauma scenarios, such as frontal or lateral impacts in MVCs or sideways falls. Given the fact that the areas encompass rib cage dimensions, there were statistically significant correlations between all measurements except for height as shown in Supplementary Figure S7. Although insignificant, the only negative correlations were between rib cage height and the three angular measurements. One direction of future work could be to implement variation of these hull shapes in human surrogates to investigate whether this parameter is a significant driver of impact loads and injury outcomes in blunt chest trauma.

Although the purpose of this research was to provide distributions of relevant rib cage measurements, we did explore the ability of multivariate multiple regression to predict the measurements. These models revealed that demographic predictors explained 53% of the variance in the dataset on average. This is comparable to previous studies where age, sex, height, weight, and BMI explained up to 50% of variance in statistical models describing whole rib cage shape (Wang et al., 2016) or individual rib shape (Holcombe et al., 2017). While our goal was to produce age-independent measures, we did see that age was a significant predictor for nine out of ten measurements collected. However, the small coefficient values for age and low relative importance (Supplementary Table S2) suggest that age does not have a clinically significant effect on the rib cage measures in this study. Lack of clinical significance was determined by the effect of age resulting in a difference of less than 1% of the minimum value for each measurement and the fact that the change was often less than the scan resolution.

An advantage of this dataset of real-world rib cages over predictive models is the ability to obtain multiple rib cage phenotypes that exist in the population *within* a narrow range of subject demographics. Consider females of average height and weight as defined by the National Health and Nutrition Examination Survey (2014) aggregated from 2013–2016 (Data Explorer II). Using the 45th-55th percentiles of stature and weight of females from this dataset provides ranges of 1.61–1.63 m and 70.7–75.5 kg, respectively. Five subjects from this study fit those criteria, and their average age, weight, and stature were used as inputs to the regression model. A comparison of the five subjects to the regression prediction is provided in Supplementary Figure S8. As expected, the predicted values match closely to the average values. Yet, the range of values from this selection cannot be represented by these predicted values alone. For several measures, the ranges encompass a significant portion of the measure’s distribution. For example, the rib cage widths span from the 10th to 76th percentiles of the female data, and the rib 7 angle spans from the 35th to 89th percentiles of the female data. In other words, two individuals with the same demographic information can fall near opposite ends of the rib cage distributions found in this study, demonstrating why the predicted values from regression models are not sufficient.

This dataset can be applied to a variety of fields involving thoracic research. Most naturally, is its application with human body finite element models. A comprehensive understanding of the range rib cage shapes seen throughout the adult population is necessary to compare current finite element models to their target demographics. On a local scale, population-based data of rib cross sectional geometry, cortical bone thickness, and rib shape has been used to evaluate 5 HBMs and suggest ways to improve geometric correspondence to population data (Holcombe et al., 2020). This concept can be applied to whole HBM rib cages using this dataset. Further, these HBMs can be made to match multiple rib cage phenotypes from within the same demographic to explore the role holistic rib cage shape plays on injury risk. Such an analysis has been performed for average males (n = 89) in the context of MVCs (Larsson et al., 2022), but can be implemented in computational studies of falls from standing (Fleps et al., 2019), falls from height (Milanowicz and Kędzior, 2017), or behind armor blunt traumas (Roberts et al., 2007; Cronin et al., 2020) with a more robust dataset.

Detailed characterization of rib cage geometry is often necessary in clinical settings as well to properly assess and treat thoracic injuries such as flail chest (Fokin et al., 2020), or conditions such as scoliosis (Lin et al., 2020) and pectus excavatum (Lim et al., 2020). Quantifying rib cage shape in a large population enhances the ability of clinicians to evaluate how shape variation, particularly at the tails of the distributions, can affect interventions. Further, studies of the biomechanical response of the rib cage during cardiopulmonary resuscitation (CPR) maneuvers could provide better insights on target compression depth given a more robust rib cage geometry to explore (Suazo et al., 2022).

There are a few limitations to note from this study. While the sample size is greatly increased compared to prior studies, females are underrepresented in the dataset. However, 494 women were included in the final dataset, and analyses were performed per sex which should mitigate the effect this imbalance has on the results. The study population was sampled from the southern region of the United States, which has been found to have a higher prevalence of obesity compared to other regions (Myers et al., 2015; Centers for Disease Control and Prevention, 2023). This may contribute to the high BMI seen in the study population. Another limitation of this study is the fact that race/ethnicity is not accounted for in the variation. In the context of hip fracture, studies have found contradicting results regarding ethnic differences in hip axis length which is an independent predictor of hip fracture (Clark et al., 2008). Differences across ethnic/racial groups could contribute to the variation seen in the adult population and may influence injury risk. Future work should include ethnic/racial data.

The ten measurements presented in this study are biomechanically relevant and affect how the rib cage responds to load. However, there may be additional measurements that are not considered here. For example, average intercostal spacing could be measured from this dataset and may influence the structural response of the rib cage. Such data could also be of use to thoracic surgeons. A future area of interest is developing a method for classifying the different rib cage phenotypes seen from this study (i.e., “bell-shaped” vs. “narrow”) and relating it back to injury risk.

The study is also limited by the absence of costal cartilage in the data. The rib cage is connected to the sternum via costal cartilage. The costal cartilages contribute to the elasticity of the thorax and connect the ribs to the sternum either directly or indirectly. Therefore, it is an important feature to consider in the context of blunt chest trauma. The dataset could be improved by including characterization of costal cartilage geometry. Similarly, we did not attempt to capture variations in cortical bone thickness, cross sectional area, or bone mineral density which all affect how the chest responds to impact (Agnew et al., 2018; Larsson et al., 2023).

The decision to represent the sternum using a single line provided a representation of the sternum body as a single structure. As defined in this study, the sternum line was based on PCA, and the first PC explained 93.4 ± 1.4 percent of the variance in the sternum on average, indicating that the sternum line represented the entire structure well. However, it is known that the manubrium and sternal body meet at an angle. Studies have found an average sternal angle of 163.75°–165.30° in females and 162.21°–166.35° in males (Selthofer et al., 2006; Ateşoğlu et al., 2018). Like the sternal angles of the current study, neither study found a statistical difference between the sexes. Separate lines to represent the sternal body and manubrium separately could lead to a more robust description of the sternum.

While a manual data quality check was implemented to detect obvious outliers caused by segmentation anomalies, the time to complete this step was small compared to the time it would take to manually perform the analysis presented here. A *post hoc* analysis of the data revealed that the removal of outliers changed the mean of each measure by less than 1%. However, the raw data showed lower minimum values and higher maximum values than the filtered dataset, due to the presence of a few outliers. Those few outliers are unlikely to affect the overall distributions, and the decision to include the quality check when using this pipeline may depend on the specific application.

Lastly, the retrospective nature of this study prevented the standardization of patient postures at the time of scan collection. We performed rotational adjustments to minimize this variability but did not perform any adjustments to address lateral curvature of the spine. As a result, perfect left-right symmetry is not present in most scans. Local rotations to correct lateral curvature, such as those performed by Wang et al. (2016) may increase the accuracy of the results.

In summary, we have demonstrated that automated segmentation and measurement extraction can be used to quantify the variation in rib cage shape seen in the adult population. We produced a dataset of over 1700 individuals encompassing this variation in rib cage geometry which can be used in a variety of applications related to biomechanics. The direct comparison of our results to prior studies on rib cage variability is challenging due to variations in measurement techniques and datasets. Nonetheless, general trends, such as the correlation between increased rib cage width and weight, or the low explanatory power of demographic parameters, echoed similar findings in the literature.

## Data Availability

The raw data supporting the conclusions of this article will be made available by the authors, without undue reservation.
