# Supplementary material for: Holistic shape variation of the rib cage in an adult population
Source: Front Bioeng Biotechnol. 2024 Sep 18;12:1432911. doi: 10.3389/fbioe.2024.1432911 (PMC11445027; doi:10.3389/fbioe.2024.1432911)
Supplement: Supplementary file 1 [file Table2.DOCX]

Table S1. Regression model coefficients. Significant Coefficients are shown in bold.

| Dependent Variable | Independent Variable | Estimate | Standard Error | p-value | Relative Importance % |
| --- | --- | --- | --- | --- | --- |
| **Height**  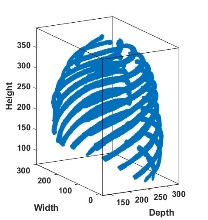 | **Intercept** | **95.84** | **9.85** | **p < 0.001** | **--** |
|  | Age | 0.16 | 0.07 | 0.0256 | 0.2 |
|  | **Stature** | **150.55** | **6.00** | **p < 0.001** | **64.7** |
|  | **Weight** | **-0.16** | **0.02** | **p < 0.001** | **2.6** |
|  | **Sex** | **10.42** | **1.28** | **p < 0.001** | **32.5** |
| **Width**  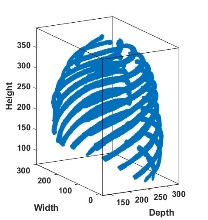 | **Intercept** | **175.66** | **8.91** | **p < 0.001** | **--** |
|  | **Age** | **0.45** | **0.07** | **p < 0.001** | **3.1** |
|  | **Stature** | **21.72** | **5.43** | **p < 0.001** | **19.0** |
|  | **Weight** | **0.61** | **0.02** | **p < 0.001** | **54.3** |
|  | **Sex** | **19.08** | **1.16** | **p < 0.001** | **23.6** |
| **Depth**  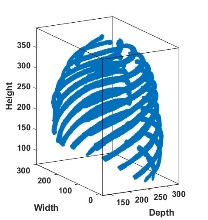 | **Intercept** | **92.20** | **7.13** | **p < 0.001** | **--** |
|  | **Age** | **0.45** | **0.05** | **p < 0.001** | **5.2** |
|  | **Stature** | **20.34** | **4.35** | **p < 0.001** | **18.5** |
|  | **Weight** | **0.46** | **0.02** | **p < 0.001** | **58.5** |
|  | **Sex** | **11.04** | **0.93** | **p < 0.001** | **17.8** |
| **Coronal**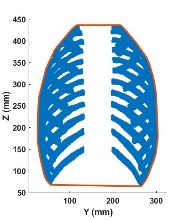 | **Intercept** | **-173.41** | **36.89** | **p < 0.001** | **--** |
|  | **Age** | **1.40** | **0.28** | **p < 0.001** | **1.0** |
|  | **Stature** | **509.29** | **22.48** | **p < 0.001** | **50.2** |
|  | **Weight** | **0.97** | **0.08** | **p < 0.001** | **14.7** |
|  | **Sex** | **72.45** | **4.80** | **p < 0.001** | **34.1** |
| **Sagittal**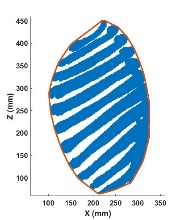 | **Intercept** | **-130.18** | **22.91** | **p < 0.001** | **--** |
|  | **Age** | **1.39** | **0.17** | **p < 0.001** | **2.8** |
|  | **Stature** | **282.54** | **13.96** | **p < 0.001** | **44.8** |
|  | **Weight** | **0.86** | **0.05** | **p < 0.001** | **22.6** |
|  | **Sex** | **41.76** | **2.98** | **p < 0.001** | **29.9** |
| **Axial**  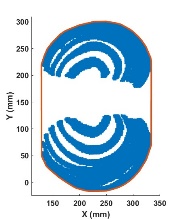 | Intercept | 54.83 | 27.90 | 0.0496 | **--** |
|  | **Age** | **1.84** | **0.21** | **p < 0.001** | **3.9** |
|  | **Stature** | **79.74** | **17.00** | **p < 0.001** | **18.2** |
|  | **Weight** | **2.21** | **0.06** | **p < 0.001** | **57.5** |
|  | **Sex** | **60.77** | **3.63** | **p < 0.001** | **20.4** |
| **Volume** | **Intercept** | **-8289.75** | **727.43** | **p < 0.001** | **--** |
|  | **Age** | **42.76** | **5.44** | **p < 0.001** | **2.3** |
|  | **Stature** | **8156.96** | **443.29** | **p < 0.001** | **38.5** |
|  | **Weight** | **38.49** | **1.57** | **p < 0.001** | **30.0** |
|  | **Sex** | **1601.89** | **94.64** | **p < 0.001** | **29.2** |
| **Rib 1 Angle**  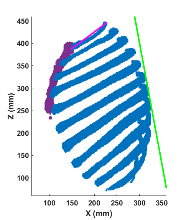 | **Intercept** | **83.84** | **3.84** | **p < 0.001** | **0.0** |
|  | **Age** | **0.12** | **0.03** | **p < 0.001** | **4.0** |
|  | **Stature** | **-30.04** | **2.34** | **p < 0.001** | **8.7** |
|  | **Weight** | **0.22** | **0.01** | **p < 0.001** | **81.3** |
|  | **Sex** | **4.73** | **0.50** | **p < 0.001** | **6.0** |
| **Rib 7 Angle**  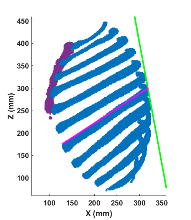 | **Intercept** | **85.77** | **3.30** | **p < 0.001** | **0.0** |
|  | **Age** | **0.11** | **0.02** | **p < 0.001** | **3.6** |
|  | **Stature** | **-29.74** | **2.01** | **p < 0.001** | **8.6** |
|  | **Weight** | **0.23** | **0.01** | **p < 0.001** | **84.1** |
|  | **Sex** | **3.99** | **0.43** | **p < 0.001** | **3.7** |
| **Sternum Angle**  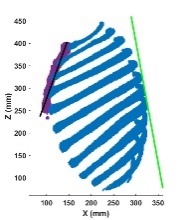 | **Intercept** | **40.44** | **2.86** | **p < 0.001** | **0.0** |
|  | **Age** | **0.06** | **0.02** | **0.0076** | **2.6** |
|  | **Stature** | **-17.67** | **1.74** | **p < 0.001** | **7.5** |
|  | **Weight** | **0.15** | **0.01** | **p < 0.001** | **88.1** |
|  | **Sex** | **1.87** | **0.37** | **p < 0.001** | **1.8** |
