# Supplementary material for: Holistic shape variation of the rib cage in an adult population
Source: Front Bioeng Biotechnol. 2024 Sep 18;12:1432911. doi: 10.3389/fbioe.2024.1432911 (PMC11445027; doi:10.3389/fbioe.2024.1432911)
Supplement: Supplementary file 3 [file DataSheet1.DOCX]

**
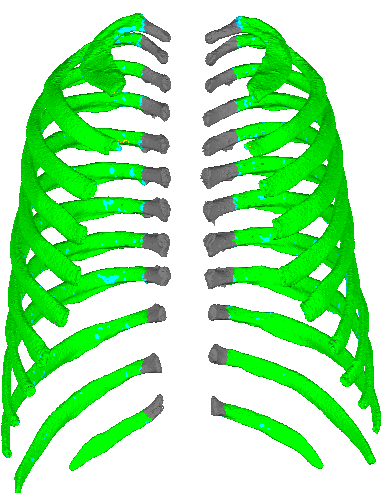
** **
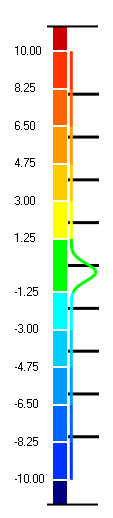
**

**Supplementary Figure S1.** Deviation of automated segmentation from manual segmentations in mm.


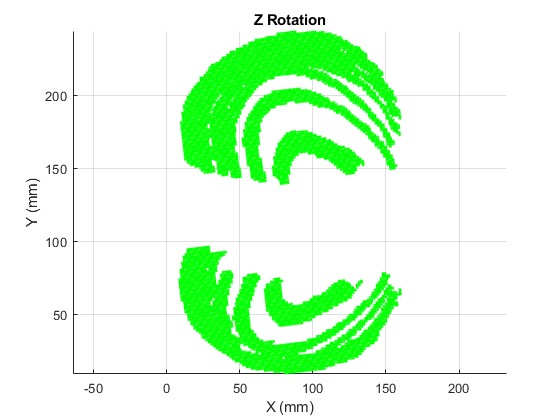

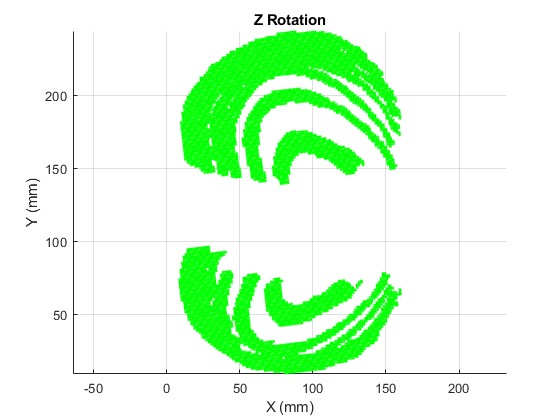

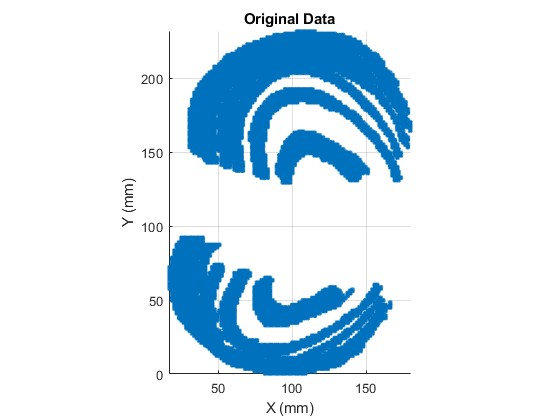


**Supplementary Figure S2.** Depiction of rotational correction applied to rib cage point clouds.

**
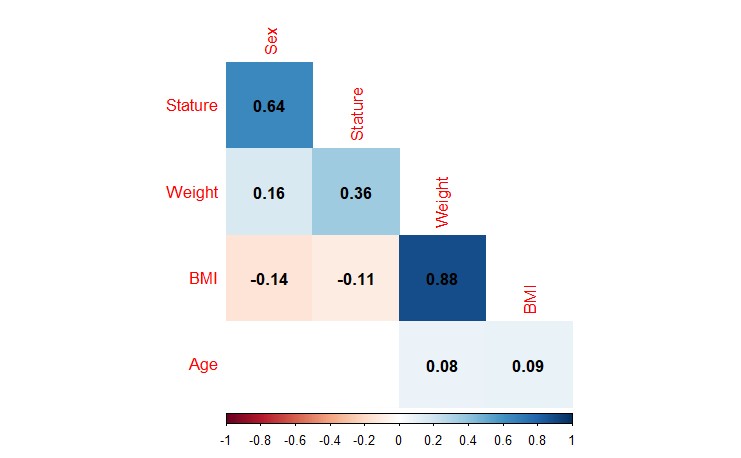
**

**Supplementary Figure S3.** Correlation plots of the five demographic predictors. Significant correlation coefficients are shown.

**Plots vs Age**

| 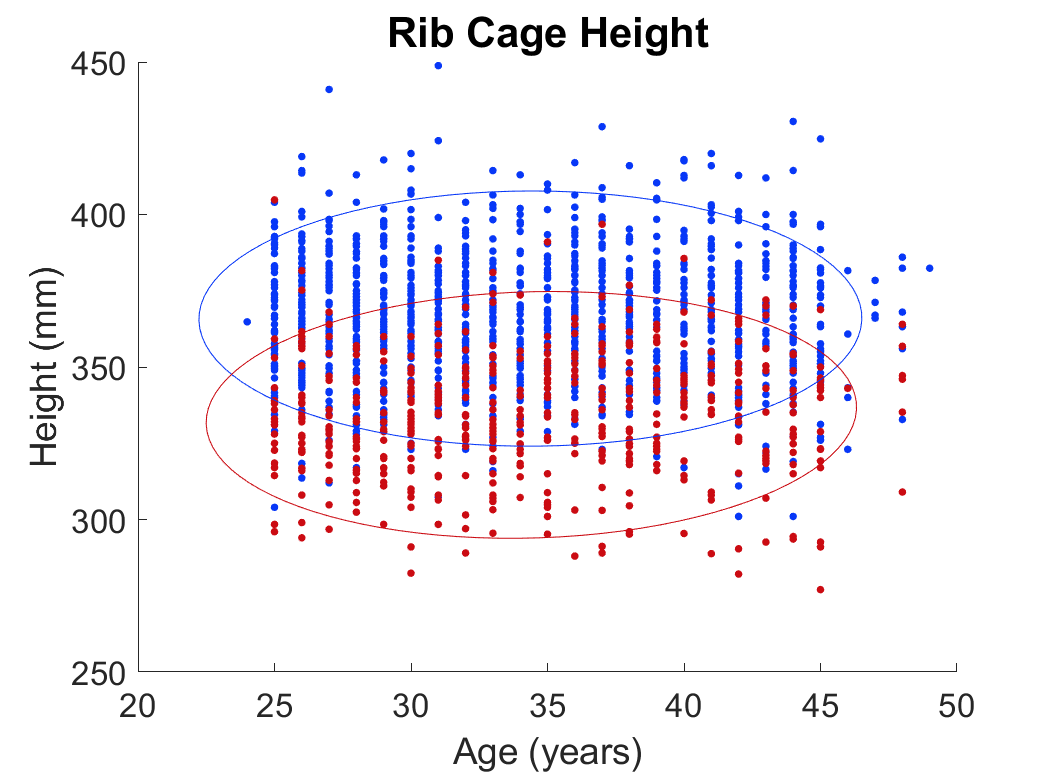 | 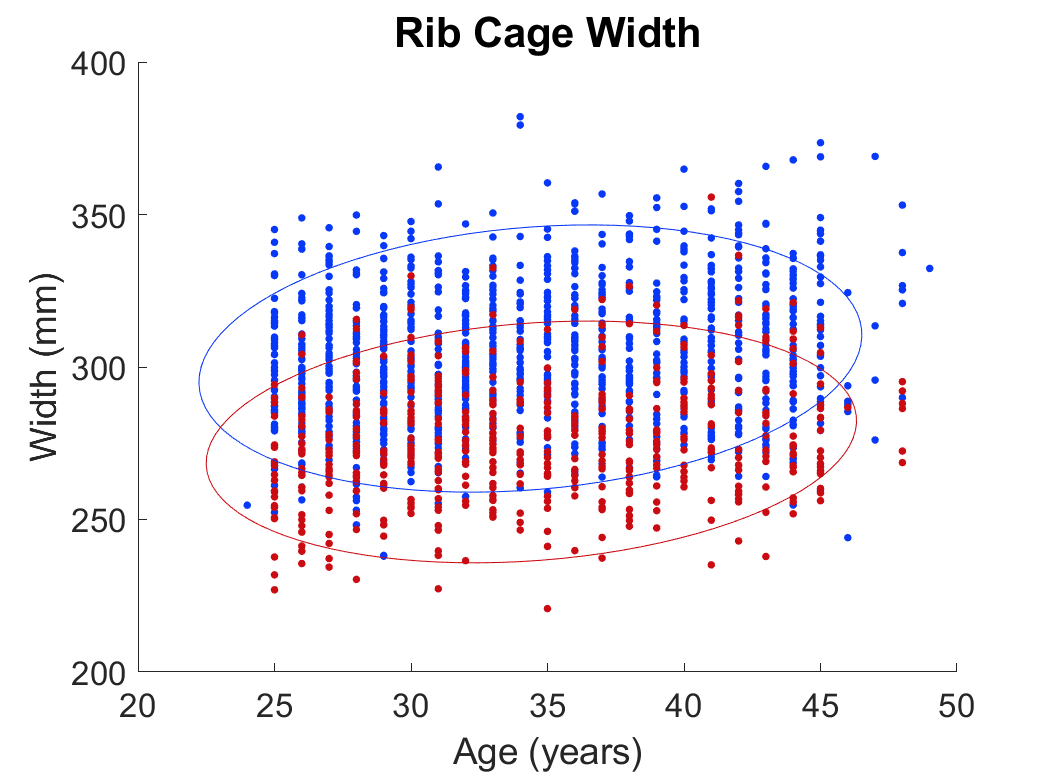 |
| --- | --- |
| 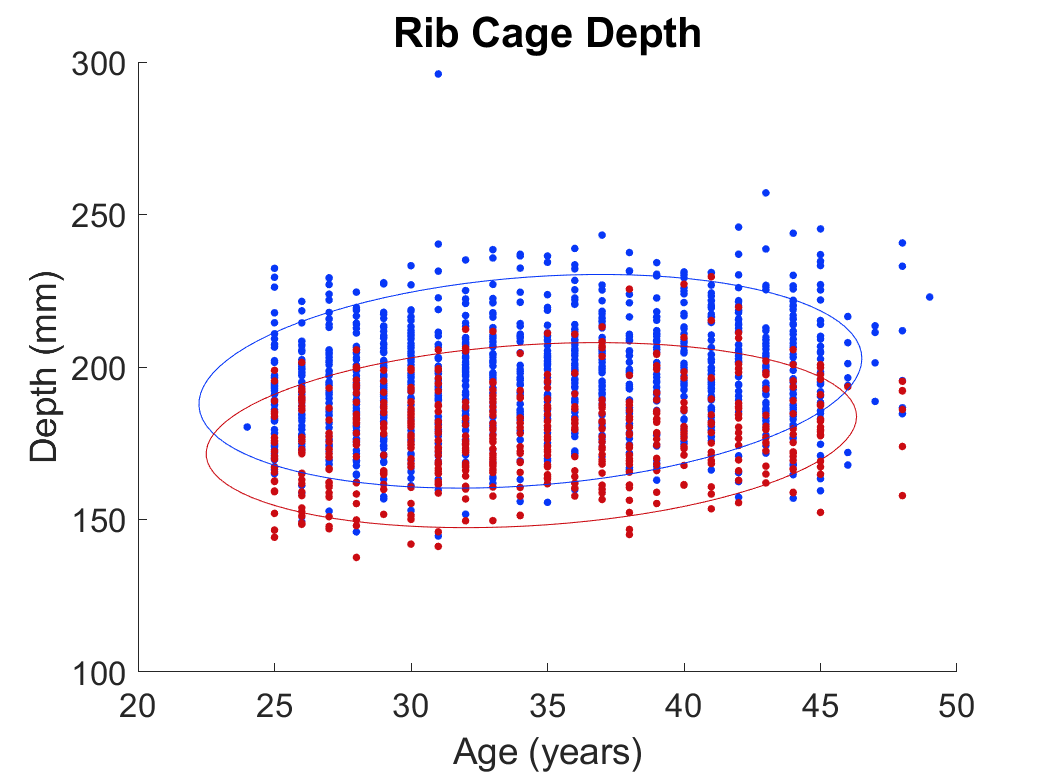 | 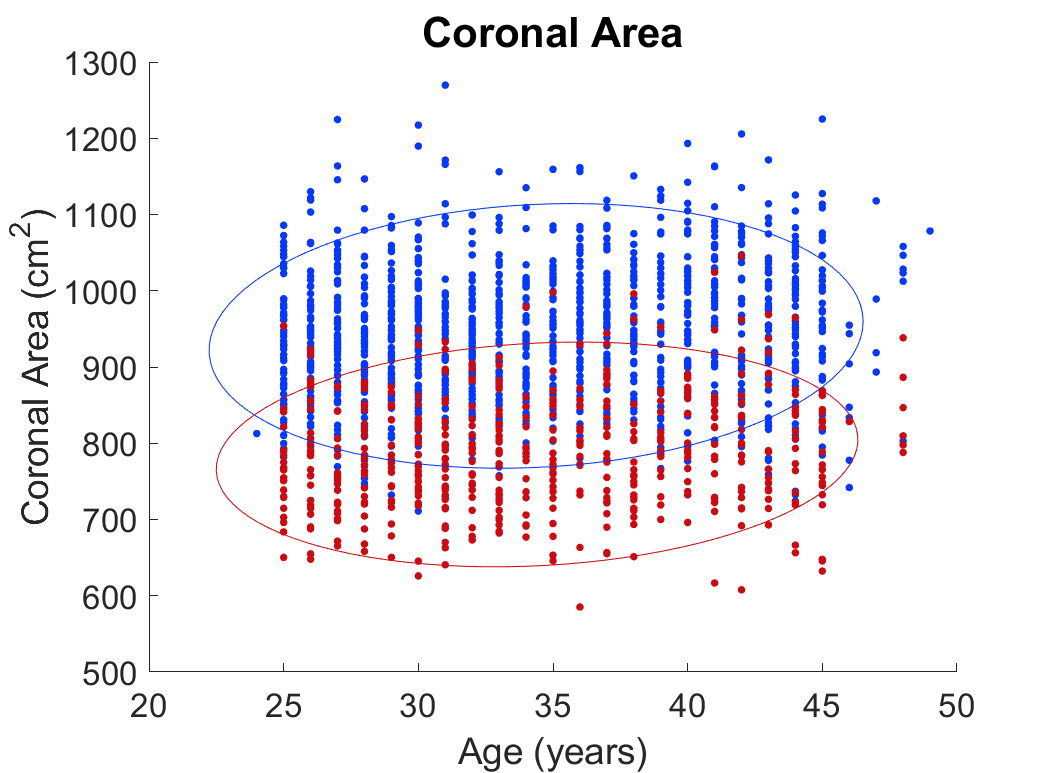 |
| 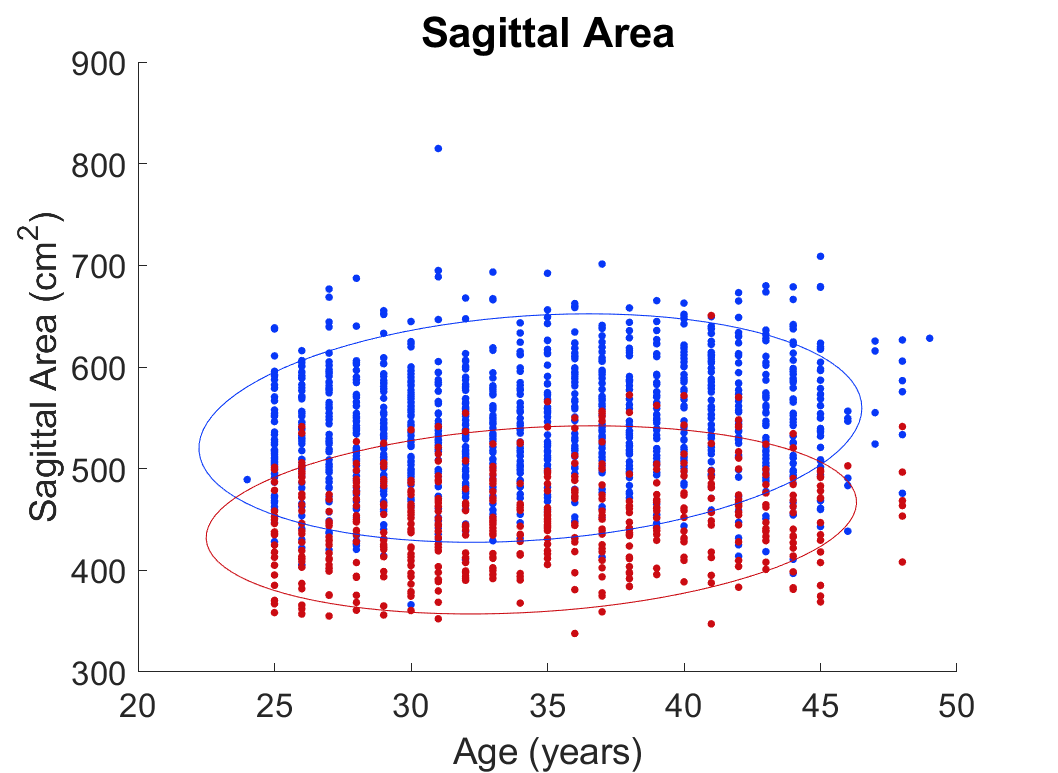 | 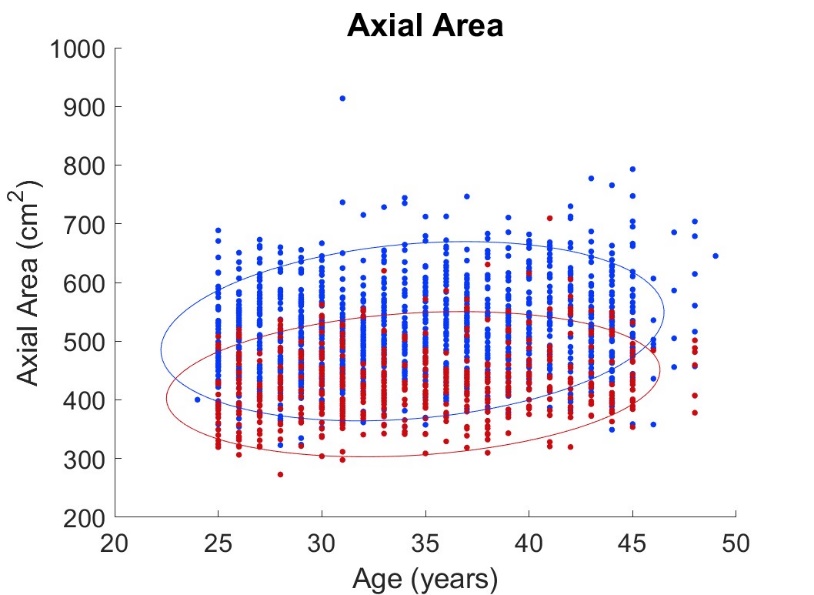 |
| 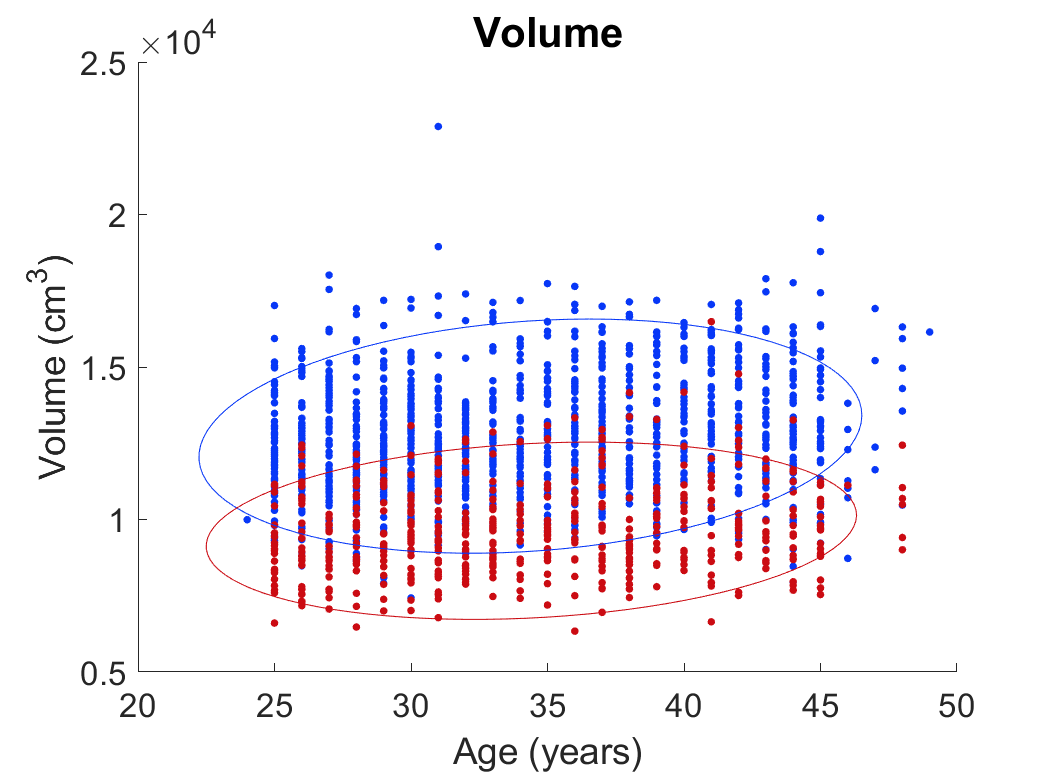 | 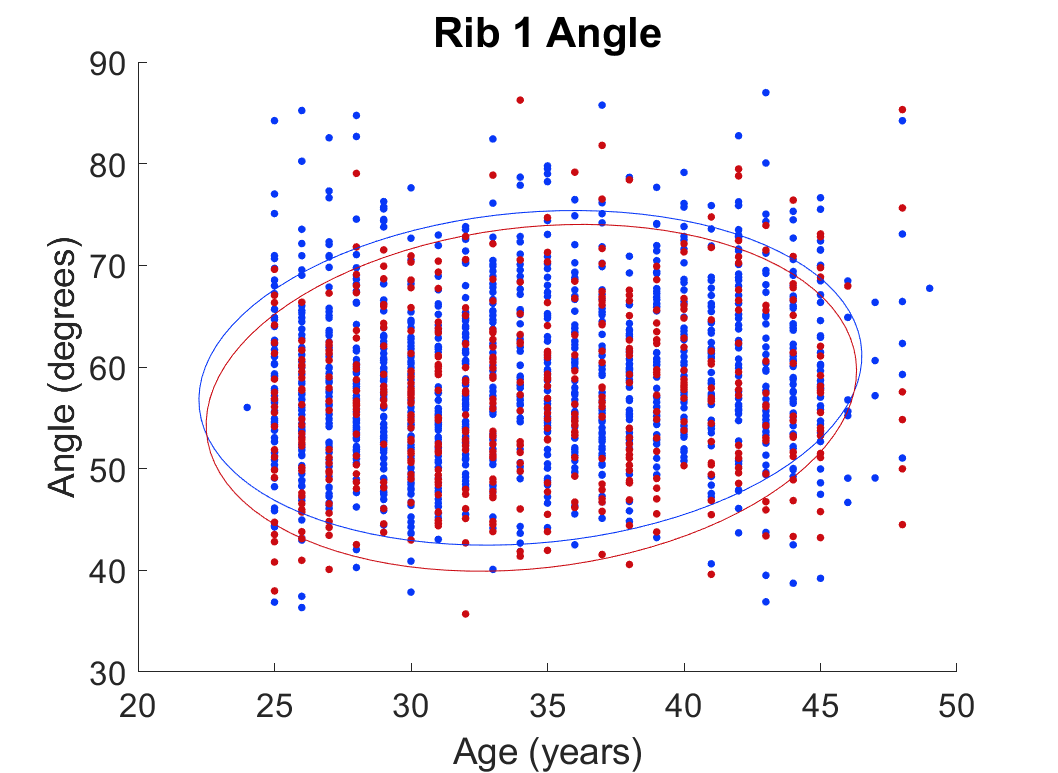 |
| 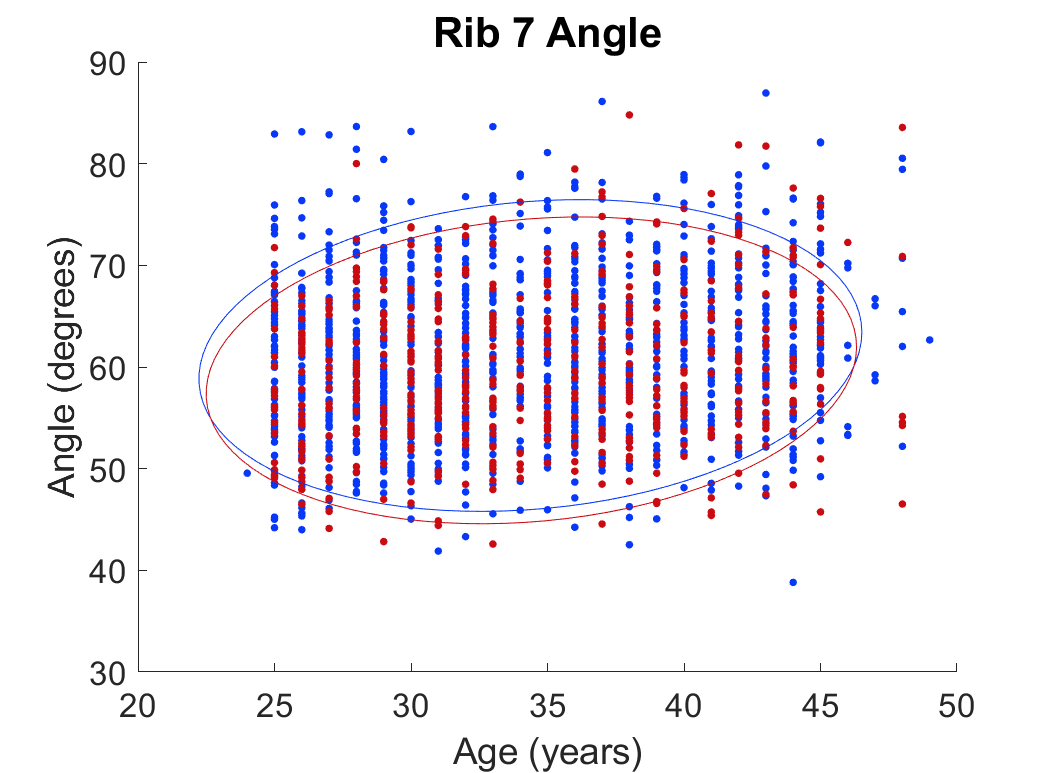 | 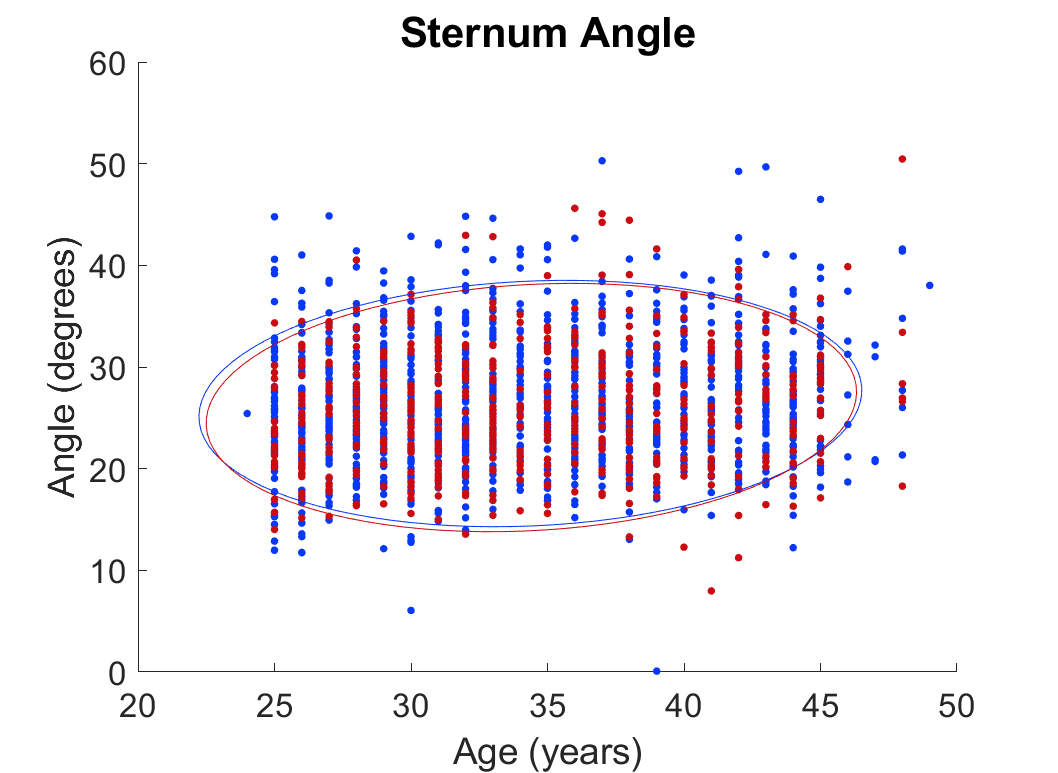 |

**Supplementary Figure S4:** Distributions of measurements vs Age.

**Plots vs Stature**

| 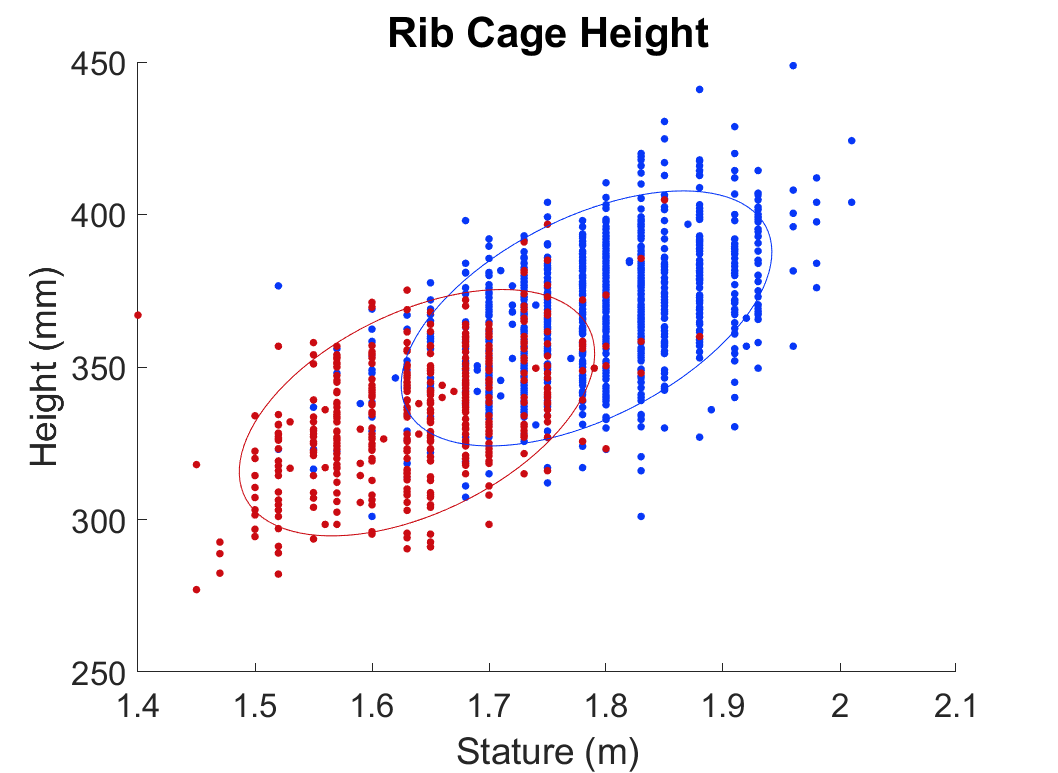 | 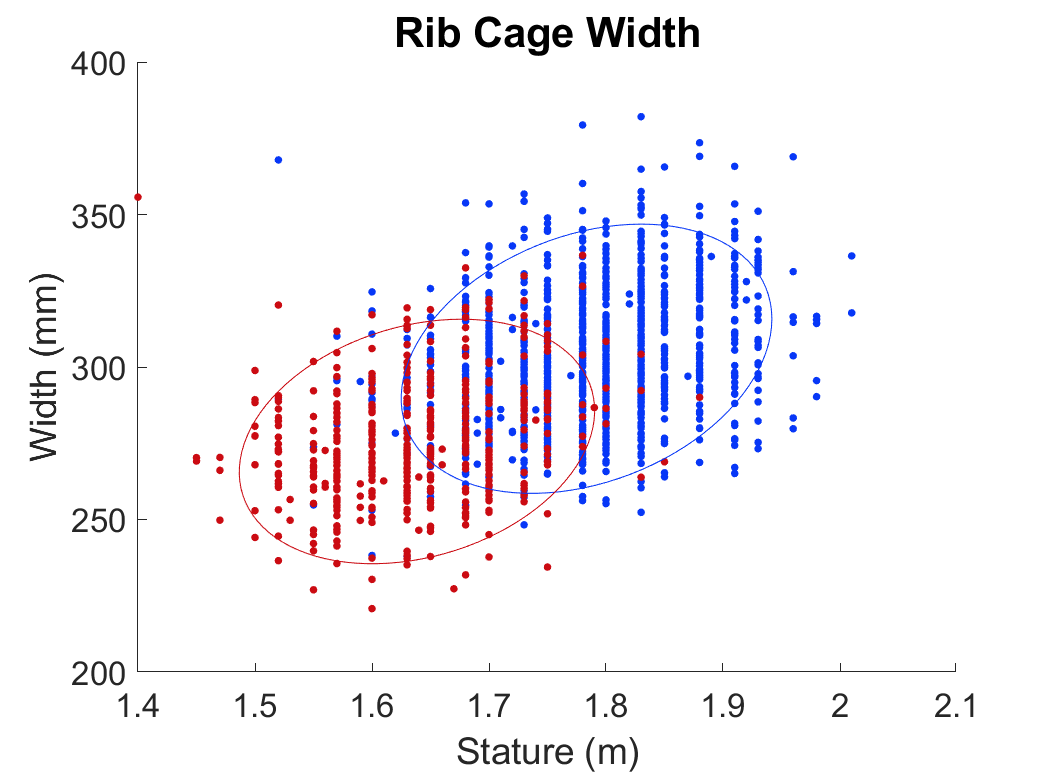 |
| --- | --- |
| 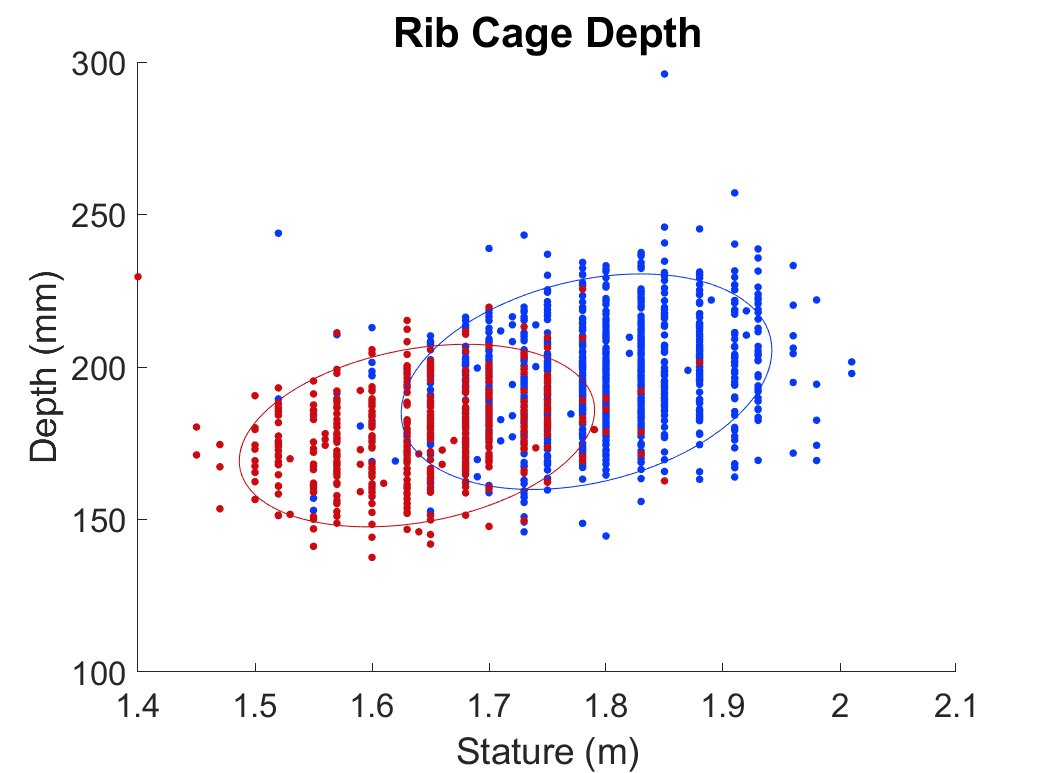 | 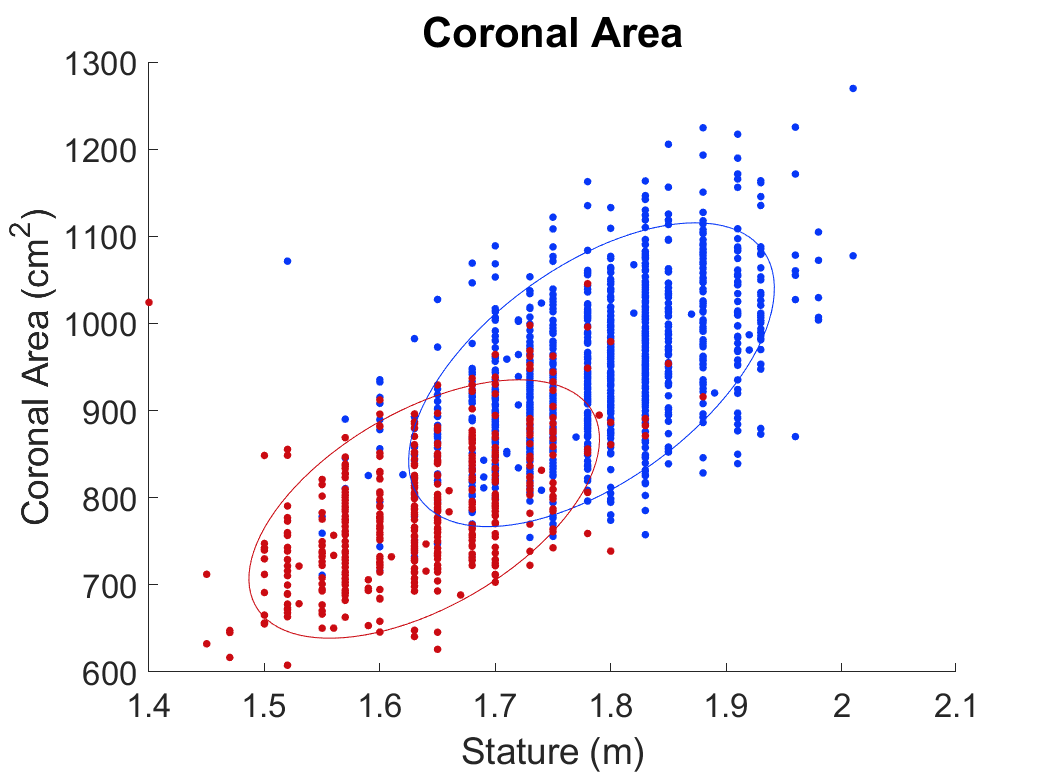 |
| 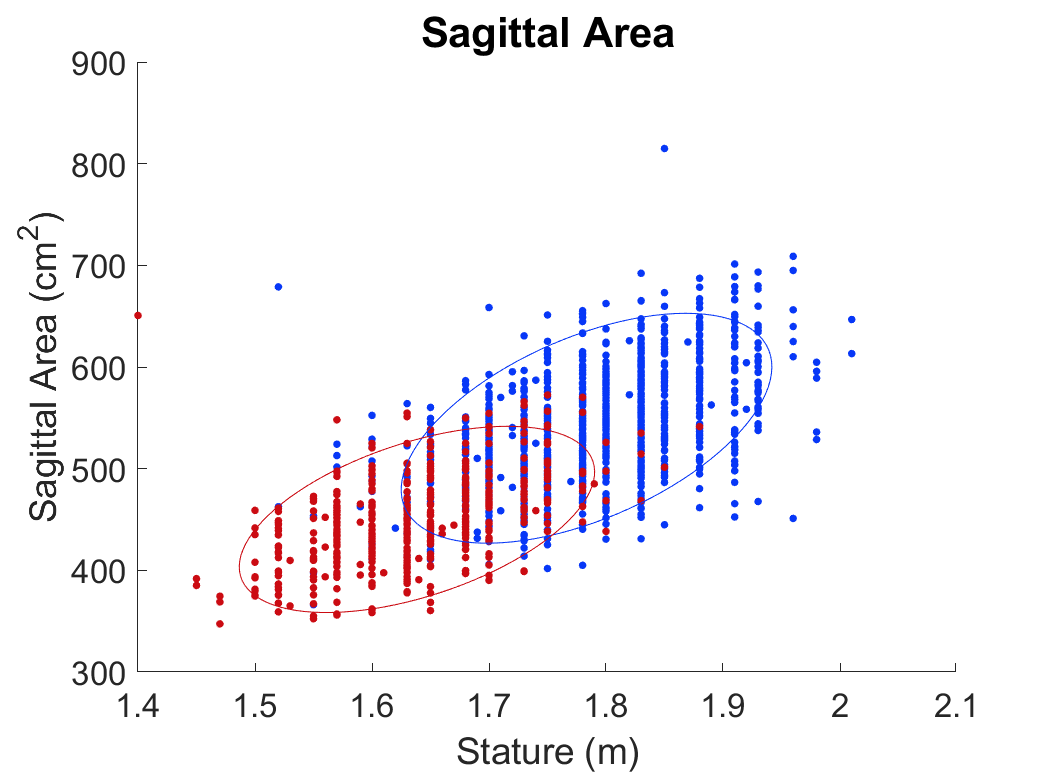 | 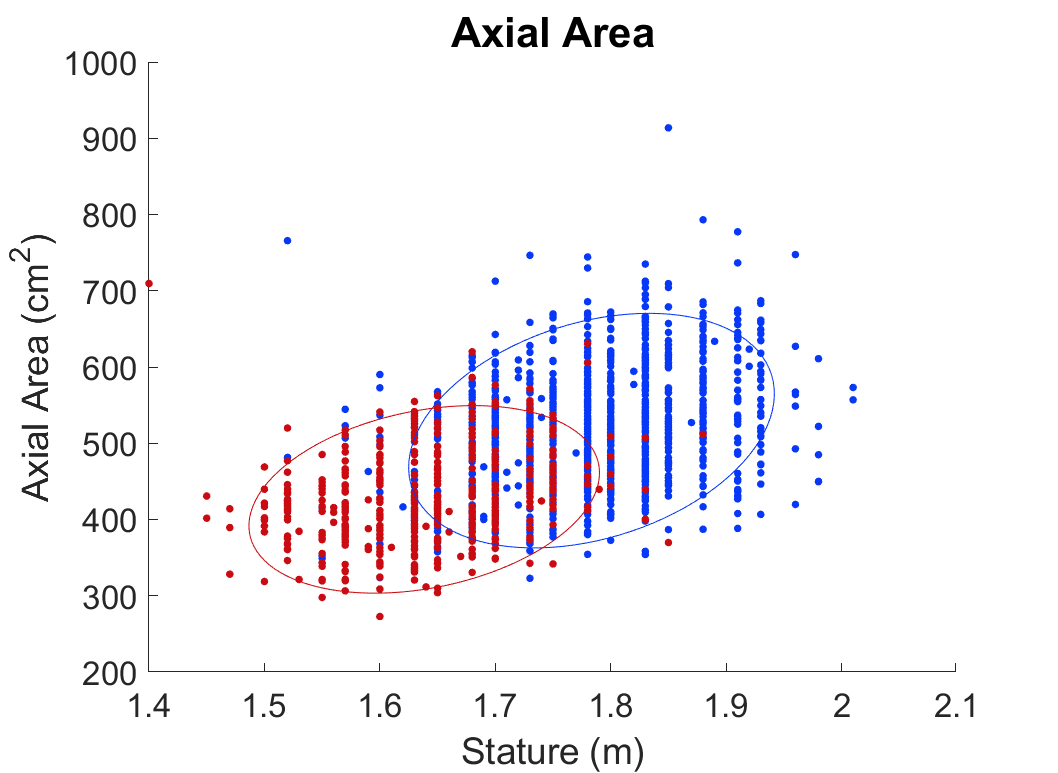 |
| 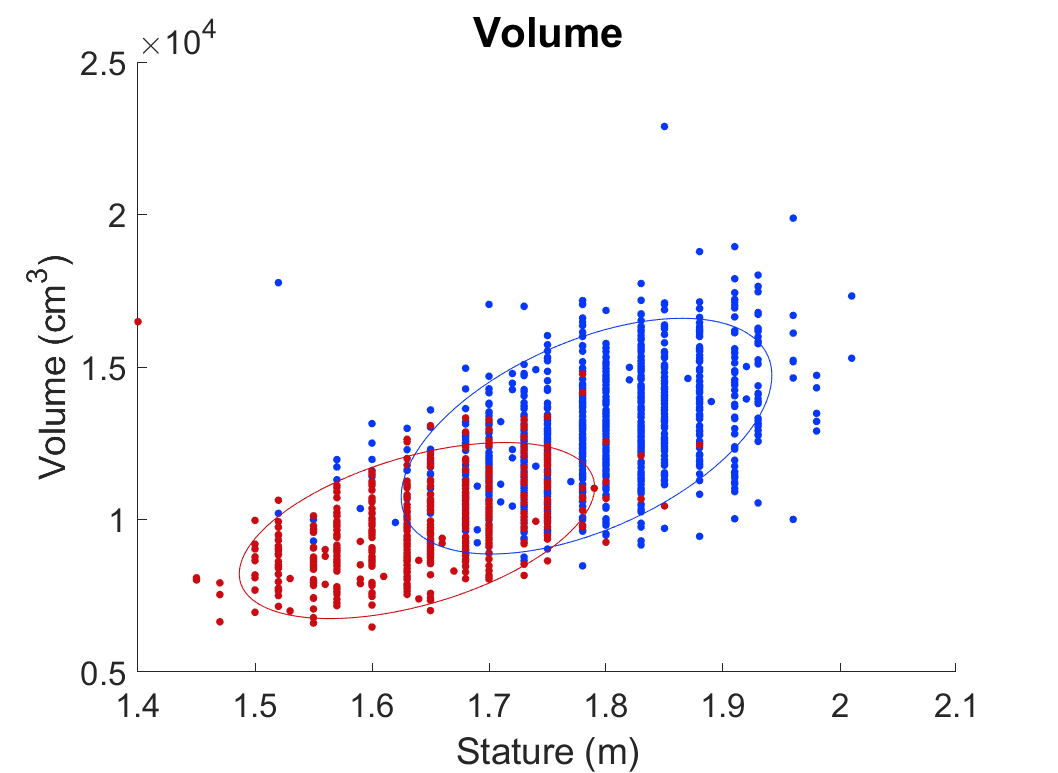 | 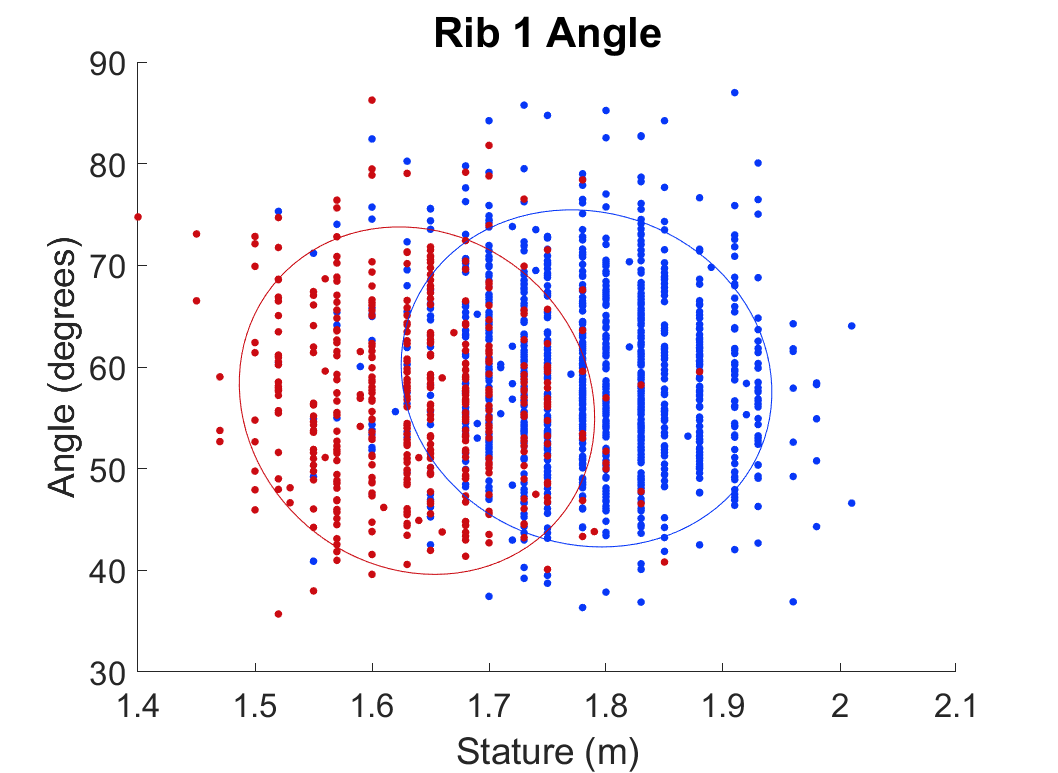 |
| 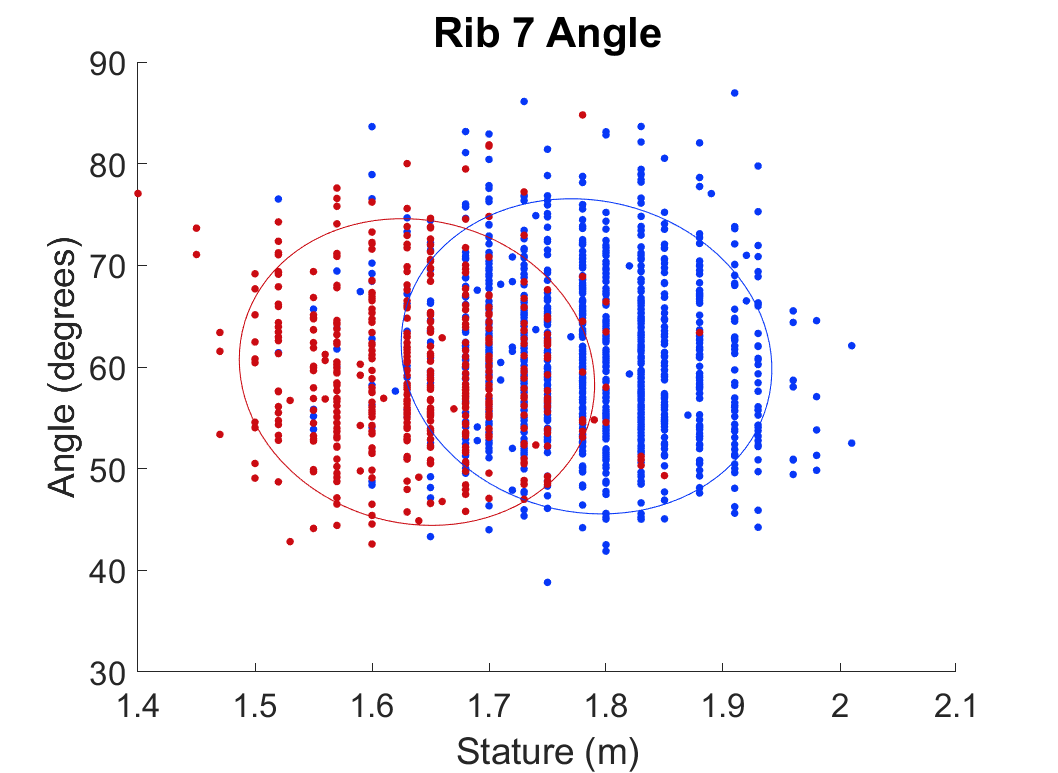 | 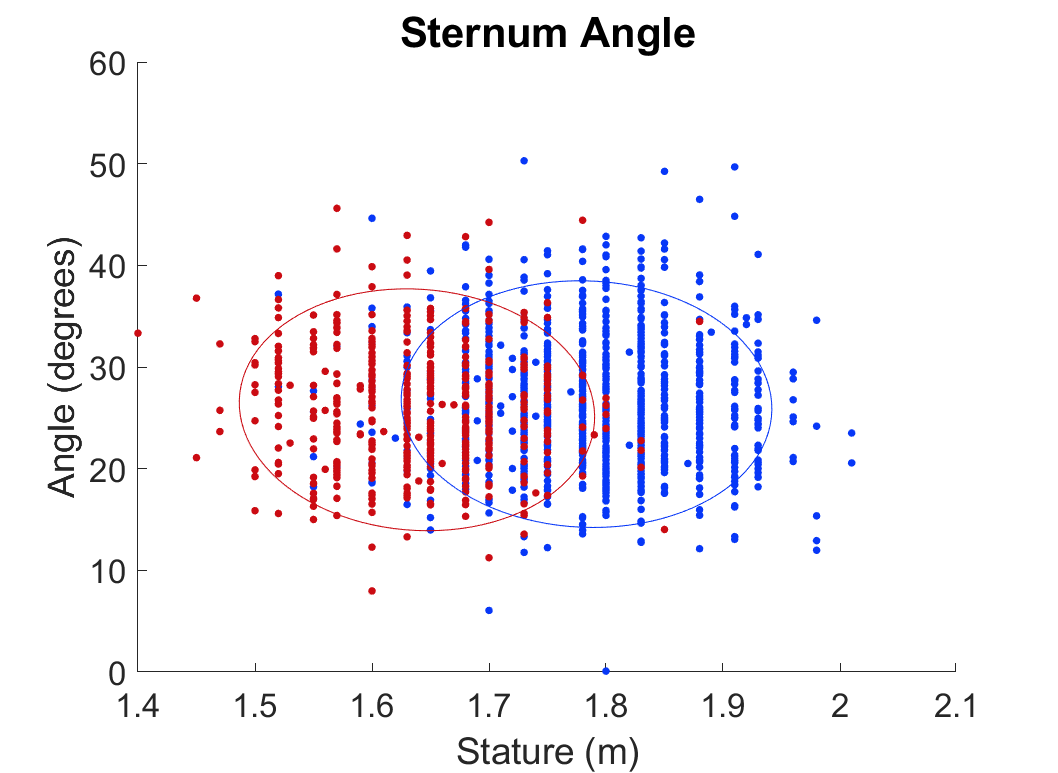 |

**Supplementary Figure S5:** Distributions of measurements vs Stature

**Plots vs Weight**

| 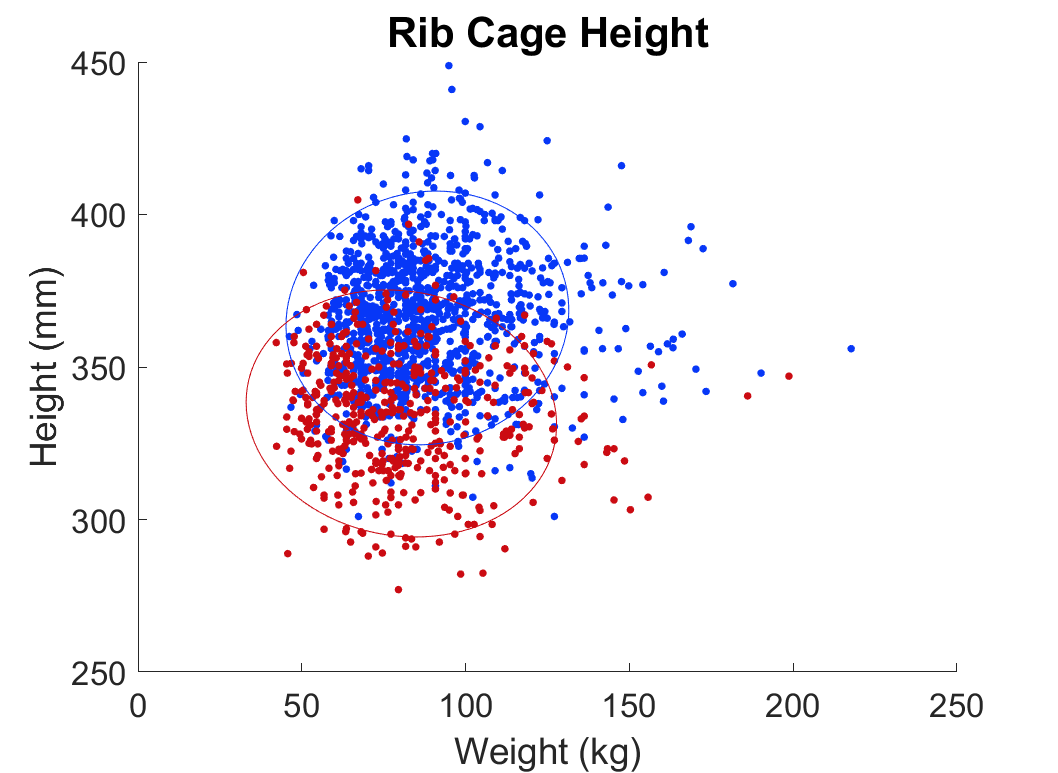 | 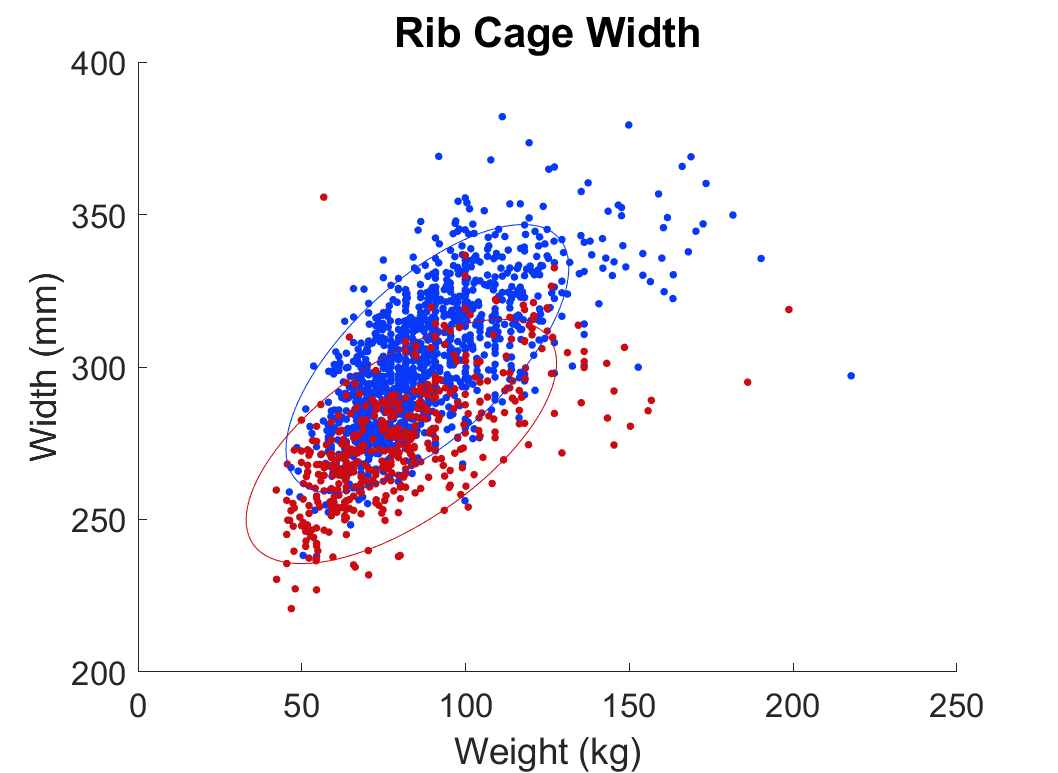 |
| --- | --- |
| 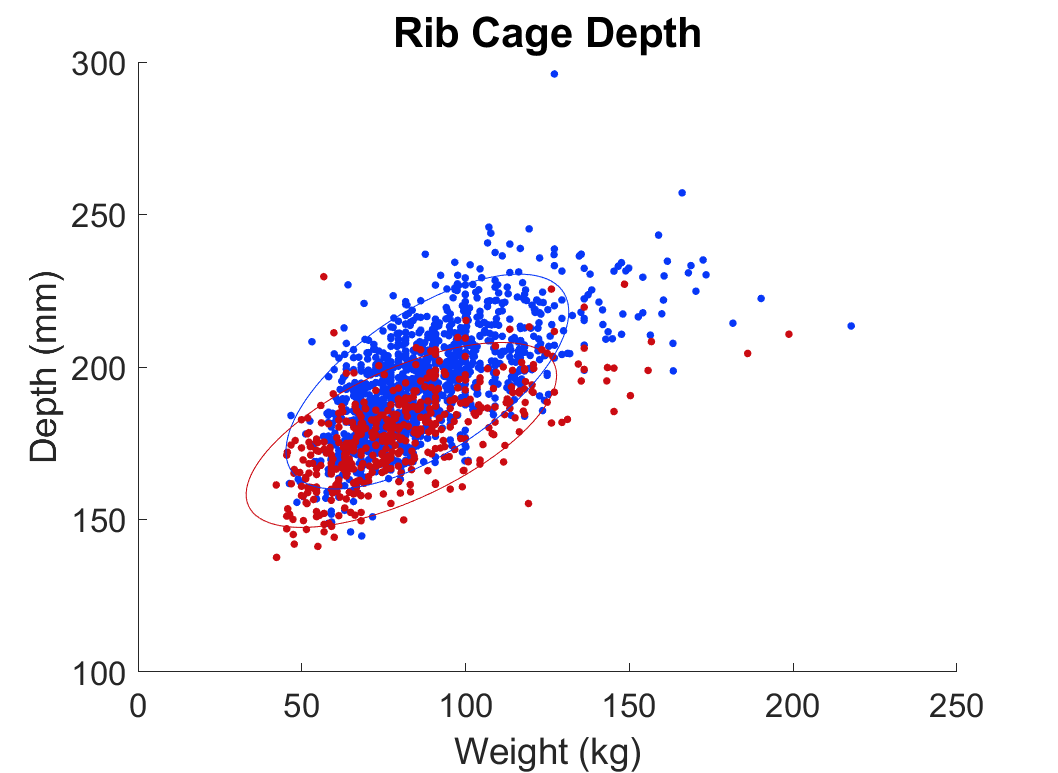 | 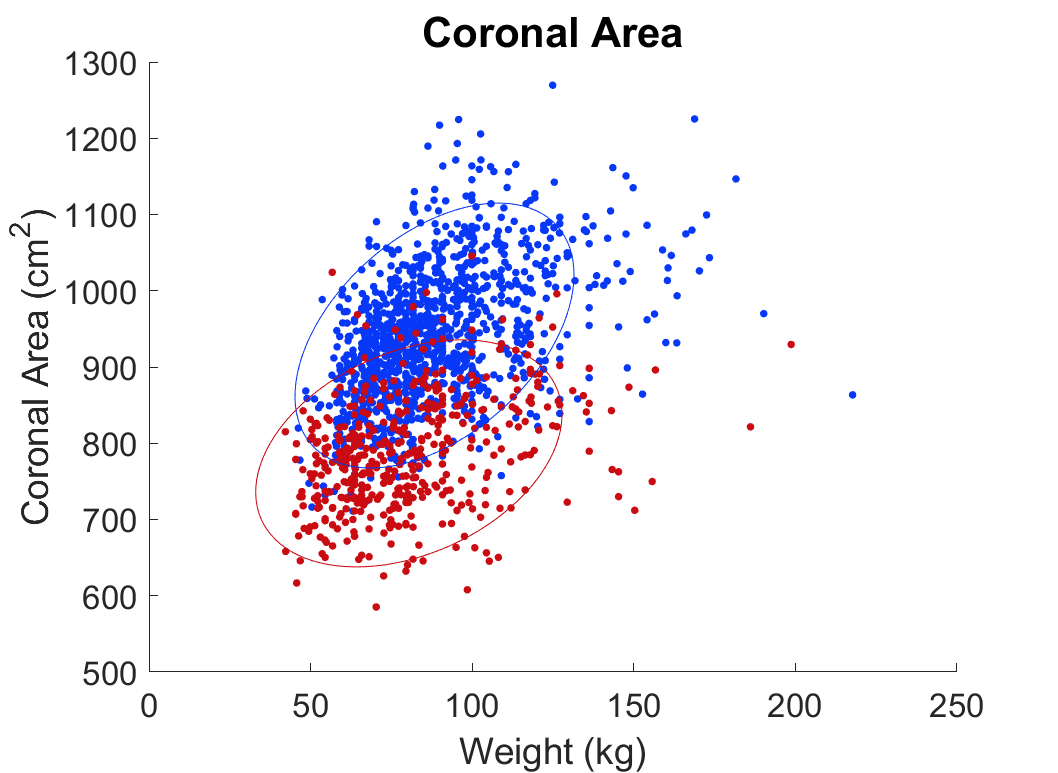 |
| 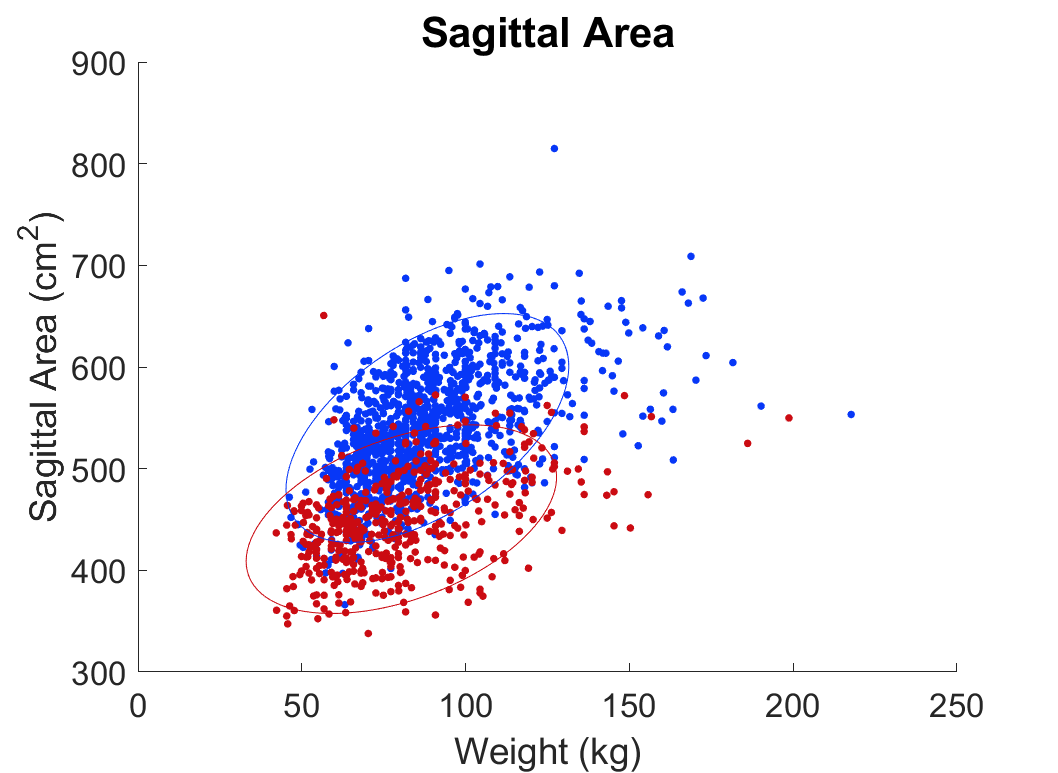 | 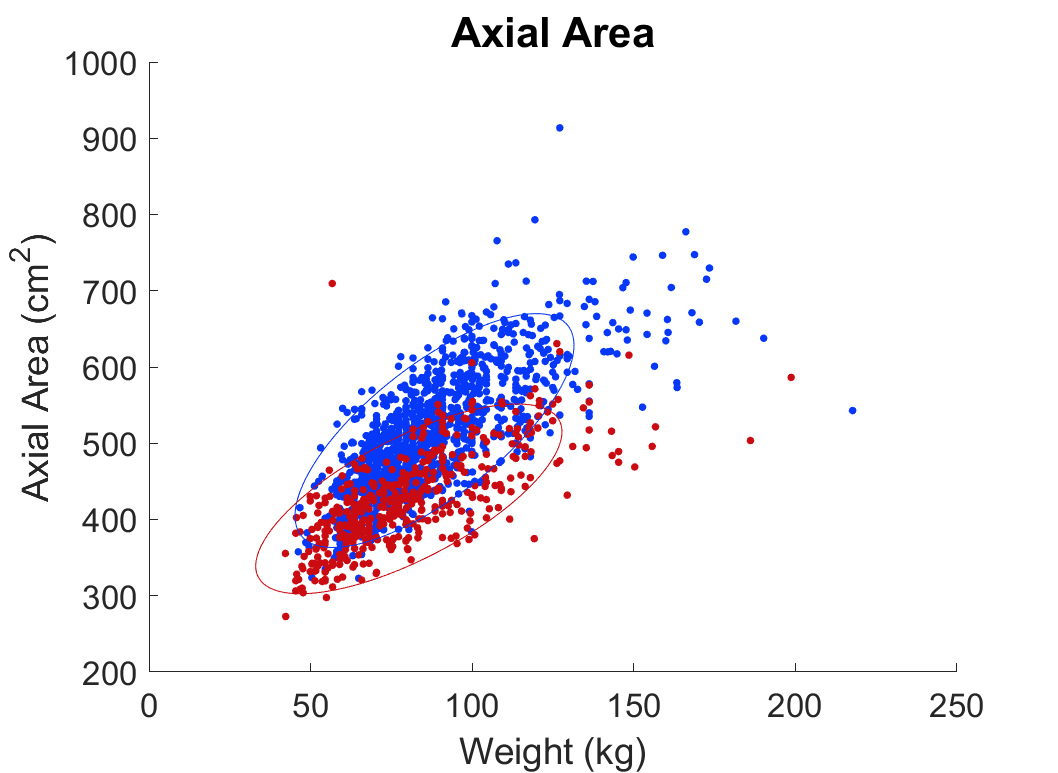 |
| 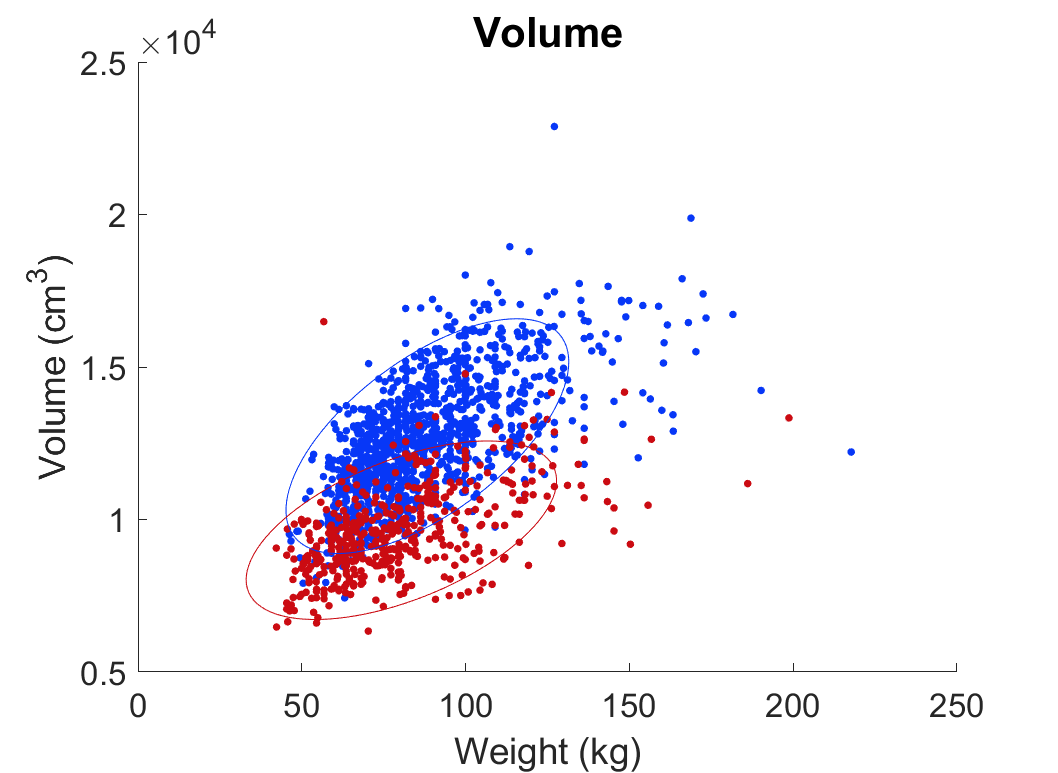 | 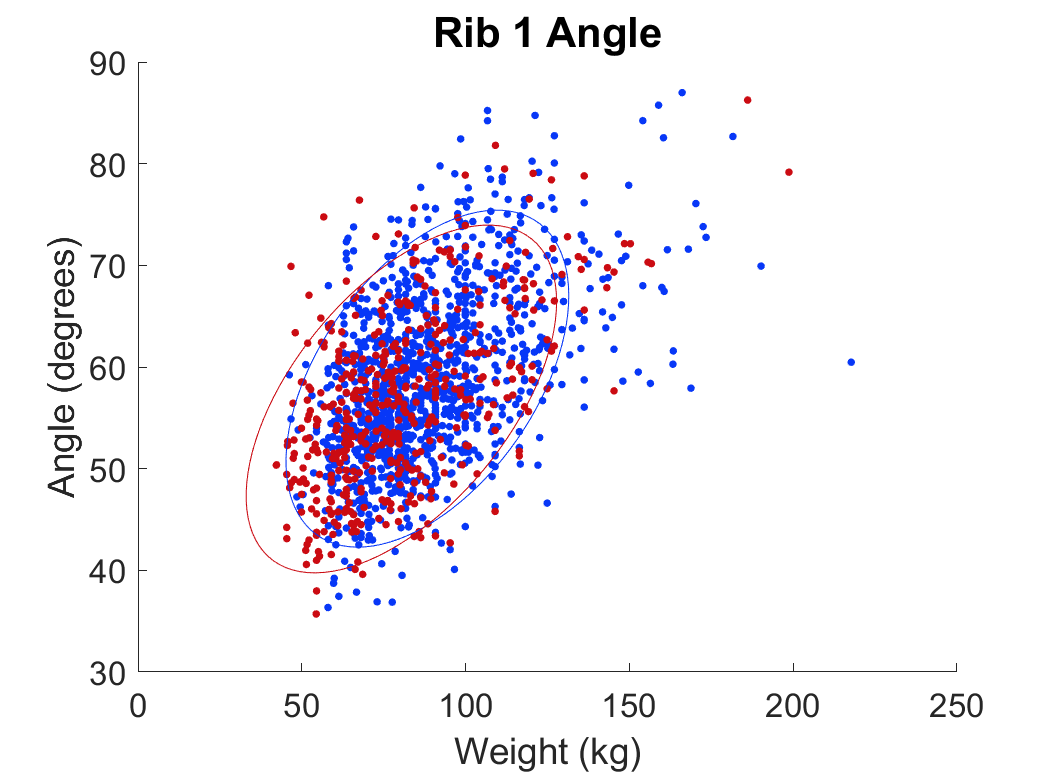 |
| 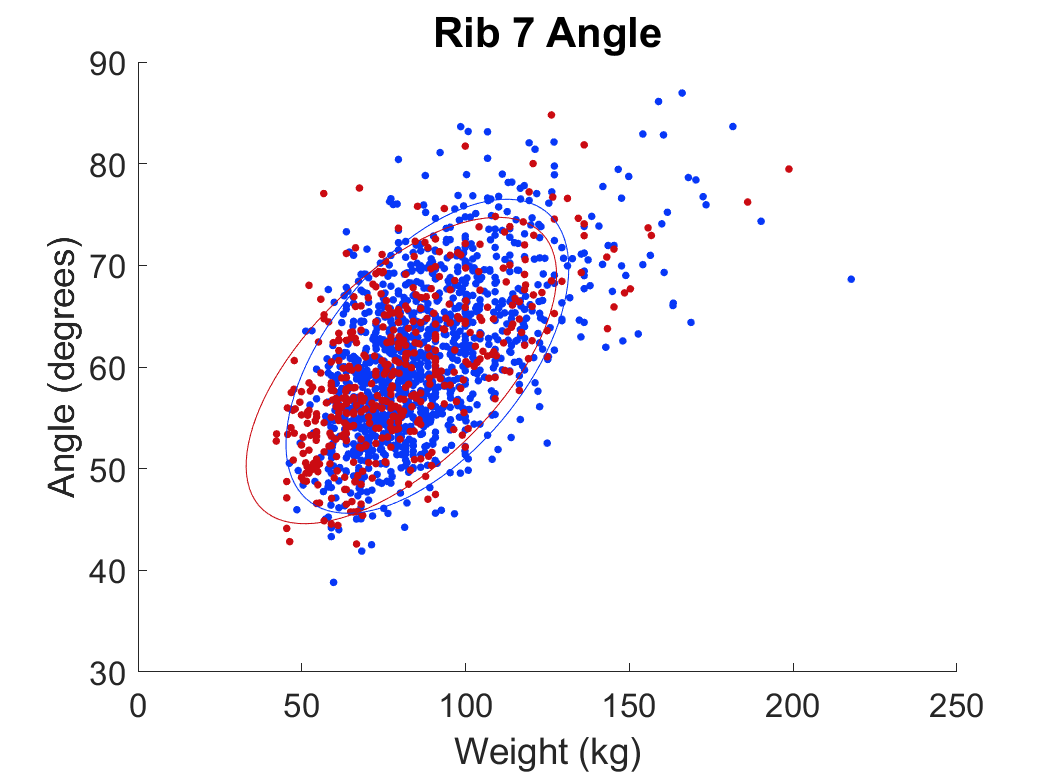 | 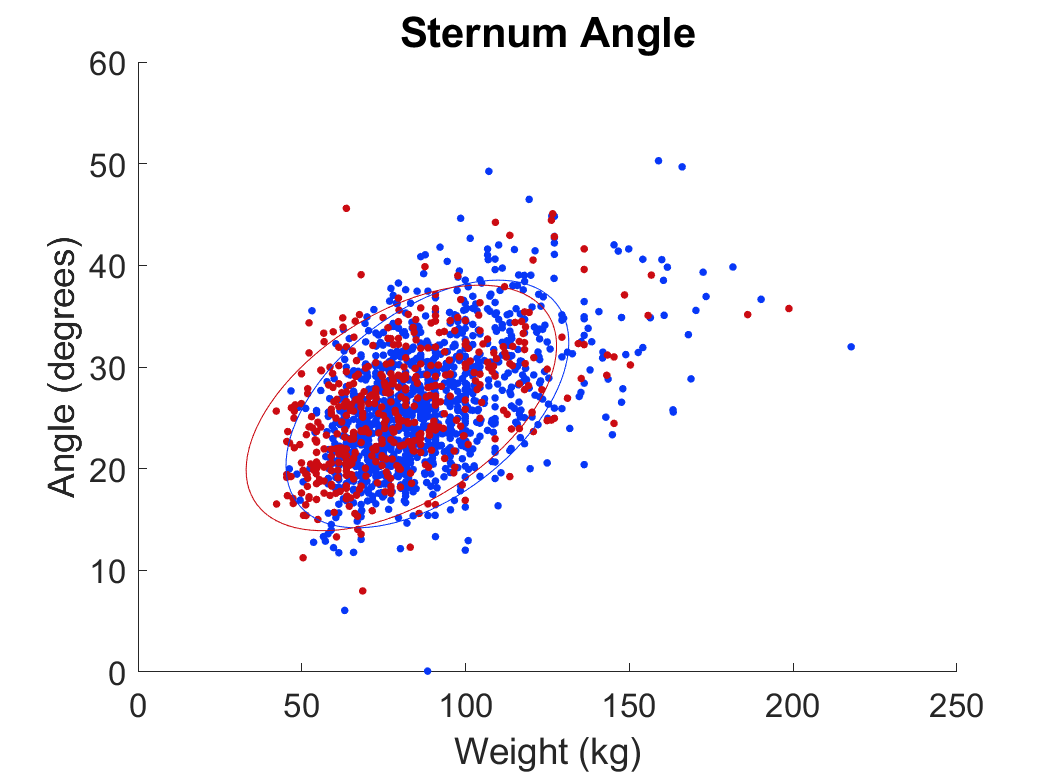 |

**Supplementary Figure S6:** Distributions of measurements vs Weight.

**
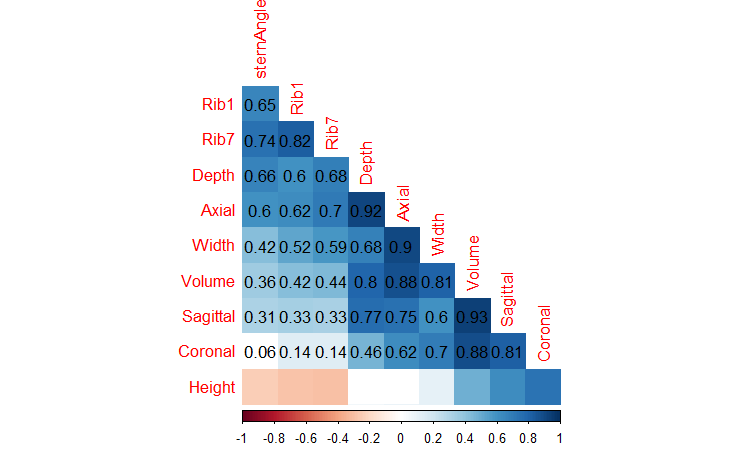
**

**Supplementary Figure S7.** Correlation plots for the ten rib cage measurements. Significant correlation coefficients are shown.

**Supplemental Figure S8.** Rib cage measurements for subjects meeting specific demographic criteria compared to the regression prediction.
